# Functional and regulatory profiling of energy metabolism in fission yeast

**DOI:** 10.1186/s13059-016-1101-2

**Published:** 2016-11-25

**Authors:** Michal Malecki, Danny A. Bitton, Maria Rodríguez-López, Charalampos Rallis, Noelia Garcia Calavia, Graeme C. Smith, Jürg Bähler

**Affiliations:** 1 Research Department of Genetics, Evolution & Environment and Institute of Healthy Ageing, University College London, London, WC1E 6BT UK; 2 Department of Genetics and Biotechnology, Faculty of Biology, University of Warsaw, Warsaw, Poland; 3 Present address: School of Health, Sport and Biosciences, University of East London, London, E15 4LZ UK

## Abstract

**Background:**

The control of energy metabolism is fundamental for cell growth and function and anomalies in it are implicated in complex diseases and ageing. Metabolism in yeast cells can be manipulated by supplying different carbon sources: yeast grown on glucose rapidly proliferates by fermentation, analogous to tumour cells growing by aerobic glycolysis, whereas on non-fermentable carbon sources metabolism shifts towards respiration.

**Results:**

We screened deletion libraries of fission yeast to identify over 200 genes required for respiratory growth. Growth media and auxotrophic mutants strongly influenced respiratory metabolism. Most genes uncovered in the mutant screens have not been implicated in respiration in budding yeast. We applied gene-expression profiling approaches to compare steady-state fermentative and respiratory growth and to analyse the dynamic adaptation to respiratory growth. The transcript levels of most genes functioning in energy metabolism pathways are coherently tuned, reflecting anticipated differences in metabolic flows between fermenting and respiring cells. We show that acetyl-CoA synthase, rather than citrate lyase, is essential for acetyl-CoA synthesis in fission yeast. We also investigated the transcriptional response to mitochondrial damage by genetic or chemical perturbations, defining a retrograde response that involves the concerted regulation of distinct groups of nuclear genes that may avert harm from mitochondrial malfunction.

**Conclusions:**

This study provides a rich framework of the genetic and regulatory basis of energy metabolism in fission yeast and beyond, and it pinpoints weaknesses of commonly used auxotroph mutants for investigating metabolism. As a model for cellular energy regulation, fission yeast provides an attractive and complementary system to budding yeast.

**Electronic supplementary material:**

The online version of this article (doi:10.1186/s13059-016-1101-2) contains supplementary material, which is available to authorized users.

## Background

Glucose is a common source of energy for cells. Glucose metabolism starts with glycolysis, which produces pyruvate. During fermentation, pyruvate is converted to organic acids, gases or ethanol. Alternatively, pyruvate can be metabolised by respiration via the mitochondrial tricarboxylic acid (TCA) cycle, also called the Krebs or citric acid cycle [1, 2]. In the mitochondrial membrane, electrons are then transferred from NADH and other TCA products to oxygen through the electron transport chain (ETC), which generates a proton gradient across the mitochondrial membrane to produce ATP by oxidative phosphorylation (OXPHOS) [1, 2]. With respect to ATP production, respiration is much more efficient than fermentation, generating a net gain of up to 36 versus only 2 ATP molecules per glucose molecule, respectively. Although respiration and fermentation share the upstream glycolysis pathway, they are to some extent antagonistic and are tuned in response to different nutrient or physiological conditions [3]. Fermentation is preferred in rapidly proliferating cells even in the presence of oxygen, a process also called aerobic glycolysis. Cancer cells, for example, typically grow by aerobic glycolysis (Warburg effect) [2]. Similarly, yeast cells proliferating in nutrient-rich media will induce fermentation and repress respiration (Crabtree effect) [4]. On the other hand, differentiated cells and yeast cells cultured in nutrient-poor media will switch to respiration [5]. Accordingly, the expression of OXPHOS genes in yeast is inversely correlated with the cellular growth rate [6, 7]. Yeast cells exhibit alternating metabolic cycles in which respiration and fermentation are temporally separated and coordinated with the cell cycle [8, 9]. Thus, respiration and fermentation are specifically tuned to environmental or physiological conditions and complement each other to support the cellular energy demands.

Cellular energy metabolism is fundamental for biological processes such as cell proliferation, stress resistance and ageing. In humans, aberrant energy metabolism results in a range of metabolic or degenerative diseases [1]. It is important, therefore, to understand the genetic factors and regulatory mechanisms that affect cellular energy metabolism. Regulation of the balance between respiration and fermentation depends largely on nutrient availability [3, 10], mediated by nutrient-sensing signalling pathways like TOR or PKA which in turn control gene expression [1, 11] as well as by direct metabolic feedback loops [12]. Moreover, it is likely that the cellular metabolic state can control gene expression or protein function via epigenetic mechanisms: the levels of key metabolites such as ATP, acetyl-CoA or NAD/NADH are readouts for energy metabolism; such metabolites can alter global levels of protein phosphorylation, acetylation, or methylation, which in turn will impact genome regulation and protein activities [1, 13, 14].

Yeasts are simple yet powerful model organisms to investigate and manipulate conserved energy metabolism programs under tightly controlled conditions by supplying different carbon sources. The budding yeast, *Saccharomyces cerevisiae*, has served as a valuable model system to study the genetic and regulatory basis of energy metabolism at a genome-wide scale [4, 5, 7, 8, 15–18]. The fission yeast, *Schizosaccharomyces pombe*, is only remotely related to budding yeast and shows features that promise valuable complementary insights into energy metabolism. Mitochondria of fission yeast form a dynamic network along microtubules which mediate their inheritance, as is the case in multicellular eukaryotes [19]. The fission yeast mitochondrial genome is compact (~20 kb, 11 protein-coding genes) and mitochondrial RNA processing is similar to in animal cells [20]. Fission yeast can grow using either respiration or fermentation but, in contrast to budding yeast, does not thrive in strictly anaerobic conditions [21]. In the presence of glucose, fission yeast grows mainly by fermentation, but it will switch to respiratory growth with glycerol [21–23] or galactose [24] as carbon sources. Unlike budding yeast, fission yeast cannot grow on ethanol because it lacks the glyoxylate cycle [25]. Although glucose represses respiration in fission yeast, this effect is weaker than in budding yeast [21], and low glucose concentrations lead to increased respiration [26]. Unlike budding yeast, fission yeast cannot tolerate the loss of mitochondrial DNA [21], which may reflect the inability to produce mitochondrial membrane potential in the absence of both the ETC and ATP synthase functions [24]. Thus, fission yeast is much more sensitive than budding yeast to mutations affecting mitochondrial functions even in the presence of glucose and many of its genes involved in respiration are essential.

Here we provide systematic analyses of energy metabolism in fission yeast using both functional and expression profiling to explore the genetic basis and regulatory processes that tune fermentation and respiration to available carbon sources. We also report novel insights into acetyl-CoA production and into the retrograde response which communicates mitochondrial damage to nuclear gene expression. These analyses provide a rich framework to inform future studies on energy metabolism.

## Results

### Genome-wide screens for genes functioning in respiration

**Fig. 1:**
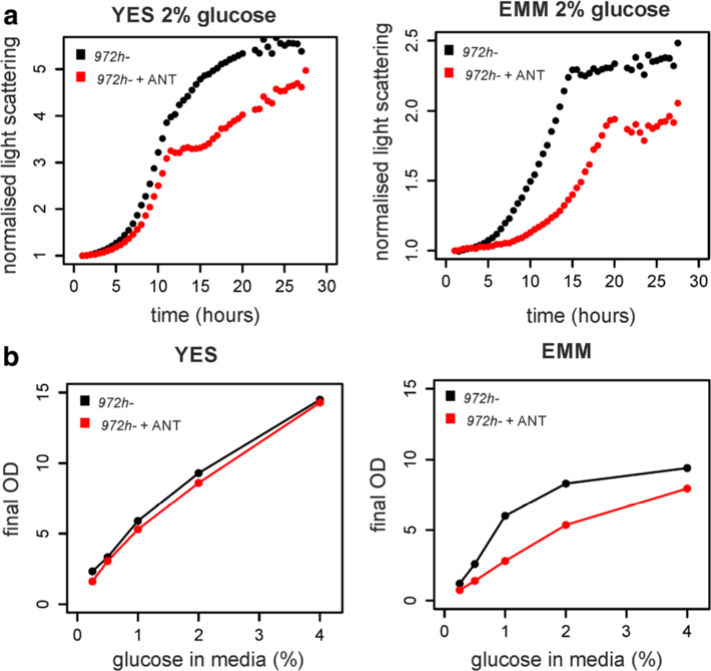
Differences in fission yeast growth media. **a** Cell growth in liquid rich (*YES*) and minimal (*EMM*) media containing 2% glucose each was measured using a BioLector microfermentor (m2p-labs), both in the absence and presence of antimycin A (*ANT*), which inhibits respiration. The graphs show averages of two repeats. **b** The same amount of cells were inoculated into either YES or EMM media supplemented with different concentrations of glucose (0.25, 0.5, 1, 2 and 4%) and the final biomass was measured by optical density (*OD*) after cells reached stationary phase. The same experiment was performed in the presence of antimycin A. The graphs show average values from two repeats for YES and four repeats for EMM

We wanted to screen for non-essential genes required for respiration using haploid *S. pombe* deletion mutant libraries. Fission yeast uses mainly fermentation when grown in abundant glucose and respiration when grown on galactose or glycerol as carbon sources [24]. Two glucose-based growth media are commonly used for fission yeast: rich YES and minimal EMM media [27]. In EMM medium, cells consume more oxygen than in YES medium [28]. We found that the respiration-inhibiting drug antimycin A slowed down cell growth substantially in EMM but much less so in YES in the same glucose concentration (Fig. 1a). These findings indicate that cells rely on respiration in EMM, even with high glucose concentration, to support their growth. Accordingly, antimycin A compromised the final biomass production more in EMM than in YES (Fig. 1b). We observed a non-linear relationship between glucose concentration and biomass production in EMM (Fig. 1b); this result suggests that another factor becomes limiting for cell growth in EMM [3]. Higher respiration of *S. pombe* cells on minimal media could affect the results of the genetic screens. For these reasons, we chose YES medium instead for the screening (see also [29]).

**Fig. 2:**
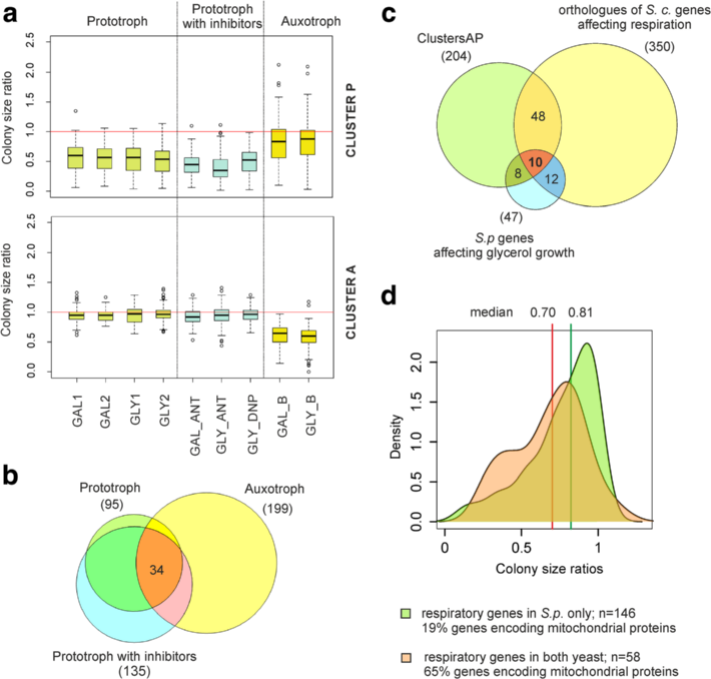
Identification of respiratory deficient mutants. **a** Fission yeast libraries of non-essential deletion mutants in auxotroph and prototroph genetic backgrounds were arrayed on solid media containing glycerol (*GLY*) or galactose (*GAL*) (respiratory conditions) or glucose (fermentative reference condition). Prototroph strains were also tested on respiratory media in the presence of inhibitors of oxidative phosphorylation: antimycin A (*ANT*) or 2,4-dinitrophenol (*DNP*). Colony size ratios between respiratory and fermentative media were calculated for each mutant. Colony size ratio distributions for clusters P and A (see main text) are presented separately as box plots. **b** Overlap between results of all genetic screens. Only mutants with colony size ratios (respiratory/fermentative media) of <0.85 were considered. **c** Venn diagram of genes from clusters A and P, genes interrogated in our genetic screens whose orthologs affect respiration in budding yeast (*S. c.*) and genes annotated to affect growth on glycerol in fission yeast (*S. p.*) (FYPO:0001934), only considering the 47 of 68 genes for which data were obtained in our screens. **d** Distribution of colony size ratios for genes that affect respiratory growth only in *S. pombe* (*green*) or in *S. pombe* and *S. cerevisiae* (*orange*); medians of the two datasets are indicated on top in *green* and *red*, respectively. The proportion of genes encoding mitochondrial proteins for the two lists is indicated at the bottom

For the genetic screening, we used high-density arrays of mutants grown on YES media plates containing glucose, glycerol or galactose as carbon sources. The original deletion mutant library has been constructed in a genetic background which contains auxotrophic mutations in *ade6, leu1* and *ura4* [30]. Such auxotrophies can significantly affect gene expression even when the required nutrients are supplemented [31]. Moreover, the *ura4* deletion is known to decrease growth on glycerol in *S. pombe* [24]. We therefore used both the original mutant library as well as a derived library in a prototroph background [32]. To identify respiratory defects, we determined the colony sizes of mutant cells grown on galactose or glycerol (respiratory media) relative to the colony sizes of mutant cells grown in parallel on glucose (fermentative medium). We performed seven screens with the prototroph library: two independent biological repeats each with mutants grown on galactose or glycerol and three screens with mutants grown on galactose or glycerol with non-lethal amounts of drugs inhibiting respiratory ATP production, i.e. the mitochondrial-uncoupling agent 2,4-dinitrophenol or the ETC inhibitor antimycin A [21]. We also performed two screens with the auxotroph library with mutants grown on galactose or glycerol. Together, these nine screens produced ratios of colony sizes on respiratory relative to fermentative medium for 2784 mutant strains in all conditions (Fig. 2a; Additional file 1: Table S1).

We applied a self-organizing map algorithm to visualise the colony size ratios from all nine screens (Additional file 2: Figure S1). Two distinct clusters contained the mutants whose growth was compromised on respiratory media, either mainly in the prototroph background (cluster P) or in the auxotroph background (cluster A). Both of these clusters were significantly enriched for genes encoding mitochondrial proteins based on Gene Ontology (GO) annotations (Additional file 2: Figure S1). The 88 mutants of cluster P showed the highest growth inhibition in the presence of respiration inhibitors and mostly showed subtle or no growth inhibition in the auxotroph background, whereas the 116 mutants of cluster A showed more growth inhibition in the auxotroph background (Fig. 2a). Only 34 mutants showed growth inhibition in both prototroph and auxotroph backgrounds (Fig. 2b). This core group comprises mostly genes encoding mitochondrial proteins and represents the most conservative hits (Additional file 2: Figure S2a). The distinct screening results from the prototroph and auxotroph libraries point to widespread genetic interactions between auxotroph markers and respiratory functions, highlighting the importance of considering effects from different strain backgrounds in genetic screens (see “Discussion”). Our combined screens using both auxotroph and prototroph backgrounds provide valuable complementary insights into the genetic basis of energy metabolism.

Among the 204 genes of clusters A and P, 18 were in common with the 47 genes annotated as affecting growth on glycerol in fission yeast and present in our experimental data [33] (Fig. 2c). These 18 genes were strongly enriched for those encoding mitochondrial proteins (Additional file 2: Figure S3). The limited overlap likely reflects differences in the mutant screens and assays used; also, most annotated genes have been identified in experiments using minimal media [26], which affect respiratory metabolism (Fig. 1). The respiratory-deficient phenotype shows high variability as noticed previously in budding yeast [34]. The results from our screens therefore provide complementary information and fresh insights.

Among the 204 genes of clusters A and P, 171 have orthologs in budding yeast. Only 58 genes of that group were in common with the 350 conserved budding yeast genes implicated in respiratory functions (Fig. 2c). These 58 genes involved in respiration in both yeasts showed overall stronger growth defects in respiratory media than the 146 genes associated with respiration only in fission yeast (Fig. 2d). The 58 common genes were highly enriched for genes encoding mitochondrial proteins (65%). We conclude that as much as ~70% of the genes identified here affect respiration in fission yeast but not in budding yeast. On the other hand, over 80% of the respiration genes identified in budding yeast were not identified in our screens. Although some of the genes associated with respiration in only one yeast may reflect experimental noise or features of the particular screens, this comparison highlights substantial differences in respiratory metabolism between the two species.

### Transcriptomes of cells growing in respiratory versus fermentative conditions

**Fig. 3:**
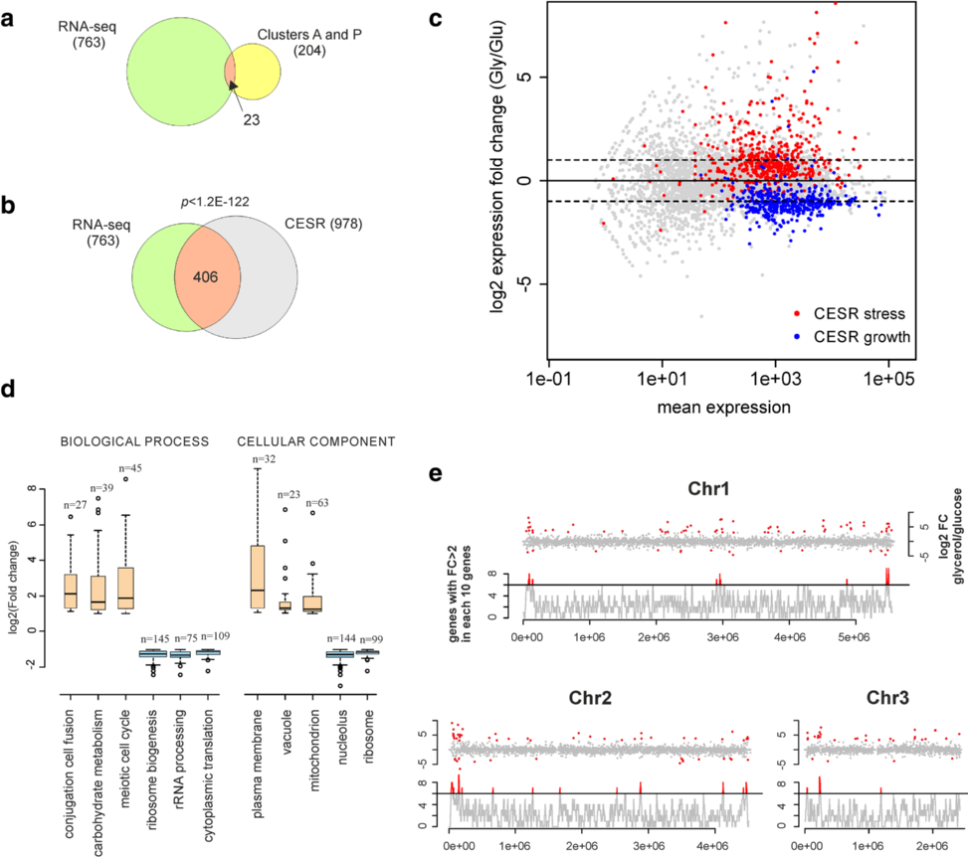
Gene expression changes in cells grown on respiratory relative to fermentative media. **a** Overlap of protein-coding genes identified by RNA-seq as significant and two-fold differentially expressed and respiratory-deficient mutants of clusters A and P. **b** Venn diagram of genes identified by RNA-seq as two-fold differentially expressed and previously defined CESR genes [35, 36]. **c** Fold changes of gene expression on glycerol relative to glucose are plotted against read numbers for transcripts in both conditions. Averages from two independent biological repeats are shown. Stress- and growth-related core environmental stress response (*CESR*) genes are highlighted in *red* and *blue*, respectively. **d** Fold-change distribution of selected GO categories overrepresented among the differentially expressed genes on glycerol relative to glucose. The widths of boxes are proportional to the number of genes in each category (n). Data obtained using AmiGO web tool (Additional file 1: Table S2). **e** *Top axes*: fold changes of gene expression on glycerol relative to glucose plotted along chromosomal gene positions, genes with log2 fold change higher than 3 or lower than −3 are shown in *red. Lower axes*: chromosomal clusters of co-regulated genes; regions with six or more genes in ten-gene windows along chromosomes being induced or repressed more than twofold are shown in *red*

We performed RNA-seq to profile genome expression in cells grown under steady-state conditions on glycerol (respiratory) or glucose (fermentative) media. In total, 763 protein-coding genes were differentially expressed between the two growth conditions applying our criteria (“Methods”): 319 genes were induced while 444 genes were repressed on glycerol compared to glucose medium (Additional file 1: Table S2). Notably, only 23 genes were both differentially expressed and required for respiration according to our screens (Fig. 3a). The differentially regulated genes also included 192 long non-coding RNAs (Additional file 2: Figure S4). Among the protein-coding genes, a significant overlap with core environmental stress response (CESR) genes was evident (Fig. 3b) [35, 36]. The CESR induced genes, enriched for cellular maintenance and stress protection, were typically induced on glycerol, whereas the CESR repressed genes, enriched for protein translation and growth, were typically repressed on glycerol (Fig. 3c). GO categories related to carbohydrate metabolism and meiotic differentiation were also enriched among the genes induced on glycerol (Fig. 3d). Consistent with the latter enrichment, cells showed efficient mating and sporulation on rich glycerol medium (Additional file 2: Figure S5), in stark contrast to rich glucose medium where meiotic differentiation is repressed. Genes encoding mitochondrial, membrane and vacuole proteins were also induced on glycerol (Fig. 3d), reflecting the enhanced respiration and changes in intracellular trafficking under this condition. Among the genes repressed on glycerol, several GO categories related to protein translation were enriched (Fig. 3d), consistent with the slower growth under this condition (Additional file 2: Figure S6).

Most of the induced genes appeared randomly distributed along the three chromosomes, but several genes near chromosome ends featured large changes in differential expression (Fig. 3e). De-repression of normally silenced regions at chromosome ends also occurs during meiotic differentiation [37]. Neighbouring genes can impact each other’s expression [38]; we therefore searched for instances where several neighbouring genes are differentially expressed. To this end, we determined the differential expression in sliding windows of ten neighbouring genes (coding or non-coding) (Fig. 3e). Besides the chromosome ends, this analysis uncovered several additional regions which contained at least seven of ten neighbouring genes that were induced or repressed (Fig. 3e). For example, the cluster at the left arm of chromosome 3 contains seven neighbouring genes that were coordinately induced on glycerol (Additional file 2: Figure S7); this cluster includes the genes encoding the high-affinity glucose transporter Ght1 and the Zwf2 enzyme for the pentose-phosphate pathway. We also noticed that at least 70% of the differentially expressed non-coding RNAs were positioned adjacent to a differentially expressed coding gene (Additional file 2: Figure S4; Additional file 1: Table S2), likely reflecting local changes in chromatin or *cis*-regulatory effects.

The co-regulated regions typically contained genes showing large changes in expression (Fig. 3e), consistent with the idea that highly expressed genes can impact their neighbourhood via chromatin changes [38]. In some cases, the neighbouring genes had related functions. The gene cluster at the right end of chromosome 2 contains three genes encoding galactose metabolism enzymes (Gal1, Gal7 and Gal10) that were strongly induced on glycerol (Additional file 2: Figure S8). Notably, growth on galactose was specifically affected in our genetic screen by deletion of the *gal1* or *gal7* genes, and in the prototroph background also by deletion of the chromatin silencing genes *cid12* and *set3* (Additional file 2: Figure S9). These results raise the possibility that *gal* genes are regulated via changes in chromatin.

### Tuning of energy metabolism

**Fig. 4:**
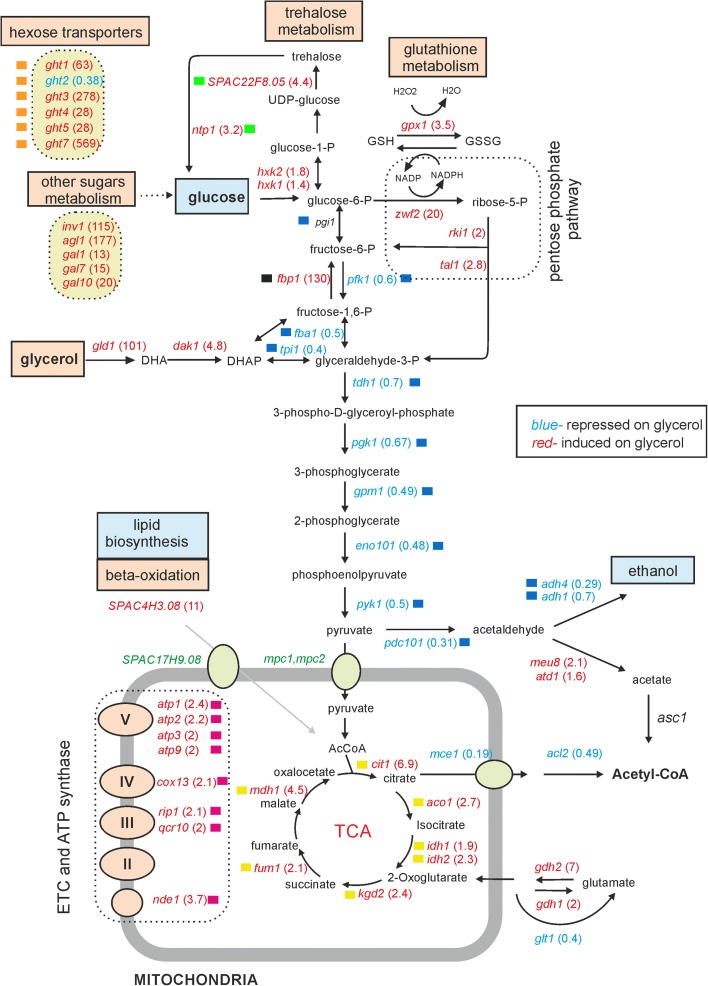
Transcriptome regulation mirrors changes in energy metabolism. Differentially expressed genes (Additional file 1: Table S2) mapped onto main energy metabolism pathways using the KEGG mapper tool. Growth on glycerol led to expression changes of carbon metabolism genes. Genes induced or repressed on glycerol relative to glucose are coloured in *red* and *blue*, respectively, with fold changes indicated in *parentheses*. Metabolites and pathways that may be up- or down-regulated, judged by gene expression changes, are highlighted in *red* and *blue*, respectively. Hexose transporters and other sugar metabolism genes are indicated on *yellow background* (*top left*). The pyruvate transporters Mcp1 and Mcp2 and the citrate importer SPAC17H9.08 (*green*) are mentioned in the text, but their expression levels were not significantly altered. *Coloured squares* indicate selected genes whose expression profiles after a shift to glycerol are shown in Additional file 2: Figure S13

Our expression profiling data provide clues about regulatory changes in energy metabolism as a function of different carbon sources. Coordinated induction or repression of sets of genes functioning in the same pathway point to up- or down-regulation of the corresponding metabolic routes. Coherent groups of genes encoding hexose transporters and enzymes involved in metabolising sugars other than glucose showed the strongest induction on glycerol (Fig. 4). An exception was the repression of the *ght2* hexose transporter gene, which could be specialised for transport under high glucose conditions. Glucose transporter genes are also extensively regulated under low glucose concentrations [39].

In the absence of glucose, genes for the main glycolytic pathway were repressed, with genes responsible for the last steps of fermentation, conversion of pyruvate to acetaldehyde and ethanol (*adh4*, *pdc101*), showing the strongest repression (Fig. 4). On the other hand, genes encoding enzymes of the TCA cycle and OXPHOS complex components were induced, reflecting re-direction of pyruvate to mitochondria.

Glycerol enters the glycolytic pathway through the action of three enzymes, Gld1, Dak1 and Dak2 [40]; *gld1* and *dak1* were induced on glycerol, as was *fbp1*, which encodes the main gluconeogenesis enzyme which produces glucose-6-P from glycerol. Glucose-6-P could fuel trehalose metabolism, the genes for which were also induced in glycerol (Fig. 4). Trehalose serves to store glucose but is also an antioxidant; the production of reactive oxygen species and thus the risk of oxidative damage are increased on respiratory media (which was also reflected by the induction of oxidative stress genes; Fig. 3c). Glucose-6-P can also be metabolised to pyruvate via the pentose phosphate pathway, the genes for which were induced on glycerol. Respiring cells could additionally benefit from flow through the pentose phosphate pathway because the resulting NADPH fuels the reduction of glutathione, which in turn can support antioxidant protection.

Sugars are directed through glycolysis to the TCA cycle, and genes for cytoplasmic and mitochondrial enzymes (e.g., *prs5*, *ser2*, *ilv5*, *lys4*) that use intermediate metabolites of this pathway for anabolic processes were down-regulated (Additional file 1: Table S2). This reduction of anabolic pathways likely reflects the slower growth rate on glycerol (Additional file 2: Figure S6) and thus decreased demand for biomolecule synthesis. Conversely, genes for enzymes that direct metabolites into the TCA cycle were induced, like those encoding glutamate dehydrogenases (Gdh1, Gdh2), which convert glutamate into the TCA cycle intermediate 2-oxoglutarate, or SPAC4H3.08, which is probably involved in beta oxidation to restore acetyl-CoA from fatty acids (Fig. 4). Taken together, our data reveal coherent transcriptome changes that mirror the metabolic rewiring under steady-state respiratory and fermentative conditions.

### Acetyl-coenzyme A metabolism

**Fig. 5:**
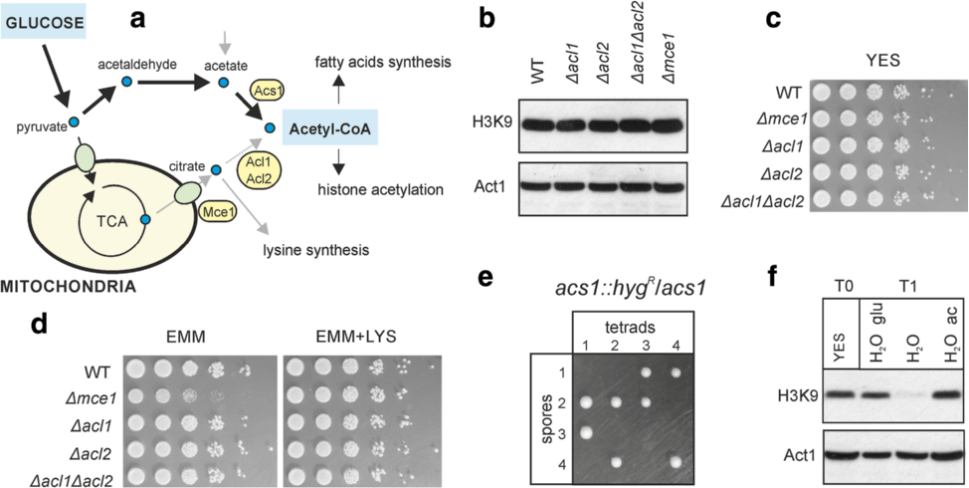
Acetyl-CoA synthesis pathways. **a** Two possible pathways that link glucose metabolism with acetyl-CoA synthesis. **b** Total proteins from wild-type (*WT*) cells or the different deletion mutants as indicated were tested for levels of histone H3 lysine 9 acetylation (*H3K9*), with actin (*Act1*) levels used as loading control. **c** Growth of indicated deletion mutants was compared to wild-type cells (*WT*) in YES medium. **d** Deletion of the *mce1* gene results in partial lysine auxotrophy. Growth of indicated deletion mutants compared to wild-type cells (*WT*) in EMM medium without (*left*) and with (*right*) lysine. **e** The *acs1* gene was replaced by the hygromycin marker in the diploid strain, followed by tetrad dissection on YES medium (four examples shown); all growing spore colonies were sensitive to hygromycin (not shown), revealing that *acs1* is essential. **f** Cells growing on YES media (*T0*) and then transferred to water completely lost histone acetylation after about 45 minutes (*T1*). This effect was suppressed if water was supplemented with glucose (*glu*) or acetate (*ac*). Total proteins were extracted and levels of H3K9 were tested, with actin levels (*Act1*) used as loading control

Acetyl-CoA is an intermediate metabolite that also serves as a substrate for protein acetylation, raising the intriguing possibility that it can transmit information on glucose availability to gene regulation via histone acetylation (Fig. 5a). Genes encoding the mitochondrial citrate exporter Mce1 (SPAC19G12.05) and subunit 2 of the ATP-citrate lyase Acl2 (SPAC22A12.16) were repressed on glycerol (Fig. 4). Acl2 transforms citrate into acetyl-CoA and oxaloacetate; in many organisms, including humans, this pathway may be crucial for cellular acetyl-CoA balance [41–43]. To test for links between acetyl-CoA metabolism and histone acetylation, we deleted the genes for the mitochondrial citrate exporter (*mce1*) and ATP-citrate lyase (*acl1* (SPBC1703.07) and *acl2*). None of these three deletion mutants showed any changes in steady-state histone H3 acetylation (Fig. 5b) or any growth phenotype in either rich or minimal fermentative media (Fig. 5c, d) or in rich respiratory media (not shown). These results indicate that ATP-citrate lyase activity is not required in proliferating fission yeast cells. Notably, deletion of *mce1* resulted in partial lysine auxotrophy (Fig. 5d). We propose that in rapidly proliferating cells, the exported mitochondrial citrate can be used to produce 2-ketoglutarate via activity of cytoplasmic fractions of aconitase and isocitrate dehydrogenase, and subsequently to produce lysine by the alpha-aminoadipate pathway [44].

The acetyl-CoA synthase Acs1 provides an alternative pathway for acetyl-CoA production from glucose (Fig. 5a) [45]. The *acs1* gene has been reported to be non-essential based on large-scale deletion analyses [30]. We independently deleted *acs1* and found that the deletion cells were not viable (Fig. 5e). We therefore conclude that Acs1 is actually essential and propose that this protein functions as the main enzyme for acetyl-CoA production in fission yeast. Consistent with this view, the substrate of Acs1, acetate, was sufficient to re-establish normal levels of histone acetylation under glucose depletion (Fig. 5f). It is unlikely that acetate was converted into glucose given that fission yeast lacks the glyoxylate cycle and thus cannot use acetate as a carbon source. We conclude that Acs1, but not the citrate lyase, is critical for acetyl-CoA synthesis under both respiratory and fermentative conditions.

### Dynamic gene regulation during adaptation to respiratory medium

In budding yeast, the volume and protein content of mitochondria increase on respiratory media [46]. We also observed more punctate mitochondrial patterns, suggesting extensive fission of mitochondria, in cells grown on glycerol compared to cells grown on glucose (Additional file 2: Figure S10). Given this adaptation to respiration, it seems surprising that only 63 transcripts for mitochondrial proteins were significantly induced on glycerol medium (Fig. 3d). This analysis included cells grown for several generations under steady-state respiratory or fermentative conditions; the main effects on gene regulation, however, may be transient and more pronounced shortly after the medium shift, when cells adapt to the new carbon source. Transient transcript changes in response to respiratory conditions such as stress or quiescence can lead to longer term changes in protein levels [47, 48]. To capture this dynamic transition, we used microarrays to profile gene expression before and at six time points after the shift from fermentative to respiratory medium.

**Fig. 6:**
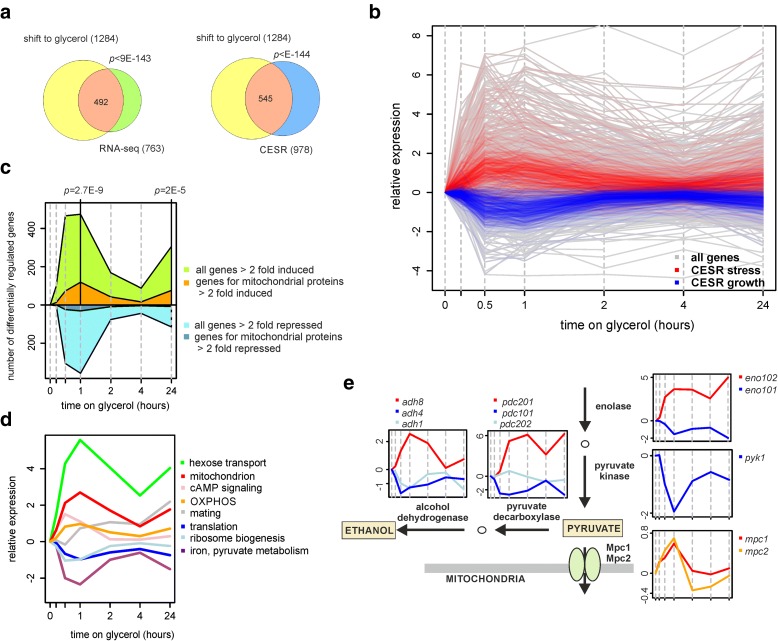
Changes in transcript levels during adaptation to the respiratory condition. Cells were grown in glucose (YES) media to early exponential phase (time point 0), when the carbon source was changed to glycerol. Transcript levels were monitored by microarrays at multiple time points after the switch (0.2, 0.5, 1, 2, 4 and 24 h). **a** Venn diagrams of genes differentially expressed more than twofold at any time point relative to time 0 after shift to glycerol, genes differentially expressed more than twofold in the RNA-seq experiment, and CESR genes. **b** Transcript profiles in response to the shift to glycerol. Expression levels relative to expression at time point 0. Transcripts for stress- and growth-related CESR genes are highlighted in *red* and *blue*, respectively. **c** Number of genes up- or down-regulated more than twofold across the time points. Differentially regulated genes encoding proteins localised to mitochondria (GO: mitochondrion) are highlighted, with significant *P* values for their enrichment among up-regulated genes indicated. **d** Transcripts were grouped into 12 clusters (Additional file 2: Figure S11). Average expression profiles for eight of these clusters are shown, along with representative enriched GO categories (see Additional file 1: Table S3 for all enriched GO categories). **e** Genes involved in pyruvate metabolism were repressed after the shift to glycerol, while isoforms of some of these genes (enolase, pyruvate decarboxylase and alcohol dehydrogenase) became induced. Plots depict expression profiles of relevant genes as indicated, along with corresponding pathways for context

This time course experiment revealed a strong response at the transcriptome level, with 1284 RNAs changing their expression levels more than twofold within the 24 h analysed (Additional file 1: Table S3). As expected, these transcriptome changes significantly overlapped with the ones detected in cells under steady-state conditions, and the response was dominated by CESR genes (Fig. 6a). CESR genes were transiently induced (‘stress-related’) or repressed (‘growth-related’) within 30 min after the medium switch, and from 1–4 h returned to expression levels similar to those before the medium shift (Fig. 6b). At 24 h on glycerol, the stress- and growth-related genes were again slightly induced and repressed, respectively, reflecting that the cells approached stationary phase by that time.

We analysed the genes that were differentially induced or repressed at each time point, including the genes encoding mitochondrial proteins. Many more mitochondrial genes became induced than repressed after the glycerol shift; this induction was somewhat delayed compared to the other induced genes, with as many as 119 mitochondrial genes peaking in expression at 1 h after the medium shift (Fig. 6c). Thus, many mitochondrial genes were up-regulated in response to glycerol, peaking in expression ~15–30 minutes after the bulk of the other induced genes. Many mitochondrial genes were then induced again at 24 h when cells approached stationary phase (Fig. 6c).

We separated all genes detected by microarrays into 12 clusters based on their expression profiles across the time course (Additional file 2: Figure S11). Each cluster was analysed for enrichments of GO categories. The clusters showing substantial expression changes were strongly enriched in categories that represent distinct metabolic functions (Fig. 6d). Clusters containing genes important for respiration (TCA cycle and OXPHOS) were induced together, peaking in expression at 1 h after the medium shift. Most notably, all of the genes encoding the ETC and the ATP synthase complex were coordinately up-regulated upon the shift to glycerol (Additional file 2: Figure S12). Moreover, genes encoding enzymes involved in carbon metabolism, which were induced or repressed under steady-state growth on glycerol (marked with coloured squares in Fig. 4), were also differentially regulated during the adaptation to glycerol (Additional file 2: Figure S13). Again, most of these genes showed the highest induction or repression at 1 h after the medium shift, and this time point therefore shows the most pronounced changes with respect to gene regulation relevant for metabolism.

Among the down-regulated clusters, one was enriched in genes functioning in iron and pyruvate metabolism. Immediately after the shift to glycerol, genes involved in pyruvate production and its transformation to ethanol were strongly repressed (Fig. 6e). Intriguingly, genes for different isoforms of enolase, pyruvate decarboxylase and alcohol dehydrogenase were induced at corresponding time points (Fig. 6e). This finding raises the possibility that the enzymes used for the last steps of glycolysis and for fermentation are replaced by these isoforms, which may have specialised functions in shifting the metabolism towards respiration. Moreover, the *mpc1* and *mpc2* genes, encoding mitochondrial pyruvate importers, were transiently induced at 1 h (Fig. 6e).

It is noteworthy that almost all the gene expression changes detected after 1 h in glycerol were largely repeated when cells approached stationary phase at 24 h. This finding suggests that the cells undergo similar metabolic changes under conditions of nutrient shortage and diminished growth. This finding suggests that the gene expression and metabolic changes during the transition to respiratory growth are similar to those during entry into stationary phase when nutrients become limiting and growth diminishes.

We conclude that during the transition from fermentative to respiratory growth the expression of many genes functioning in key metabolic pathways is strongly regulated, with maximal changes around 1 h after the shift to glycerol. Many of these genes, however, remain differentially expressed during steady-state conditions as reflected by the strong overall agreement between our RNA-seq and microarray data. Genes encoding isoforms of metabolic enzymes are often antagonistically regulated during the adaptation from fermentative to respiratory growth, raising the possibility that they have specialised roles in either growth condition.

### Communication between mitochondria and nucleus: defining a retrograde response

Mitochondrial damage can impact nuclear transcription through the retrograde signalling response [49]. In budding yeast, loss of mitochondrial DNA or chemical inhibition of the ETC lead to nuclear gene regulation in response to the mitochondrial dysfunction [50]. In fission yeast, different deletion mutants of respiratory metabolism genes show altered expression of a similar group of nuclear transcripts, suggesting the existence of a retrograde response [51, 52].

**Fig. 7:**
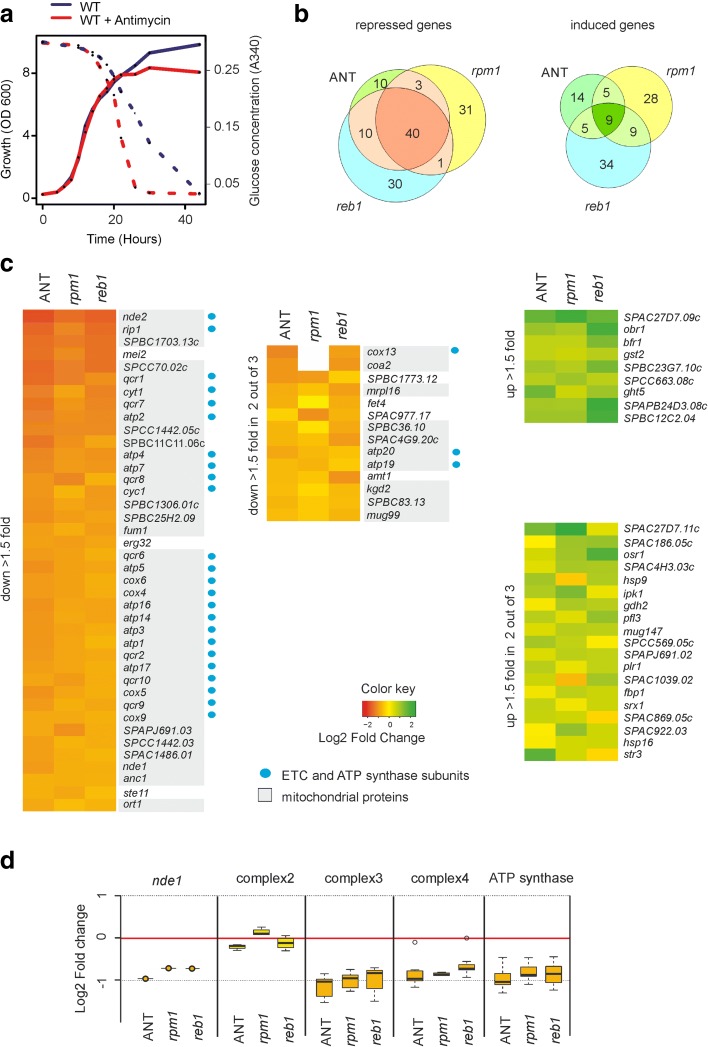
Characterisation of retrograde response in fission yeast. **a** Blocking ETC activity results in higher fermentation with similar growth rate. Growth rate curves (*solid lines*) and glucose concentration in the medium (*dotted lines*) for wild-type (*WT*) and antimycin-treated cells as indicated over time. **b** Venn diagrams of genes induced or repressed >1.5-fold in *rpm1* mutants, *reb1* mutants, and antimycin-treated cells (*ANT*). **c** OXPHOS genes are repressed in response to mitochondrial dysfunction. Heat maps showing 40 repressed (*orange*, *left*) and nine induced (*green*, *top right*) genes present in overlap from **b**. *Colour intensities* represent fold changes as in indicated in key. Additional panels show the 14 repressed (*middle*) and 19 induced (*bottom right*) genes differentially expressed in only two of three experiments. OXPHOS genes are marked by *blue dots*. **d** Genes of complex 2 are not repressed in response to mitochondrial dysfunction. Box plot showing fold changes of transcript levels encoding OXPHOS complexes and Nde1, in *rpm1* and *reb1* deletions and antimycin-treated cells as indicated

To analyse any retrograde response in fission yeast, we inhibited the ETC by antimycin A and studied cellular gene expression changes by microarrays. Cells treated with antimycin A in fermentative medium showed the same growth rate as untreated cells, but they reached a lower biomass in stationary phase (Fig. 7a). The treated cells consumed glucose at a faster rate and produced more ethanol, indicating that the energy metabolism was shifted even more towards fermentation (Fig. 7a). We analysed the transcriptomes of antimycin A-treated cells to untreated control cells during early exponential growth phase. We then compared the genes that were differentially expressed in response to antimycin A (Additional file 1: Table S4) to the genes that were differentially expressed in two different respiratory-deficient mutants, *rpm1* [52] and *reb1* (M. R.-L., unpublished data). Rpm1 is a mitochondrial RNA exonuclease involved in the processing and degradation of mitochondrial transcripts [53]; its deletion therefore abolishes production of mitochondrial-encoded ETC subunits (the *rpm1* deletion was not present in our mutant libraries for the genetic screens). Reb1 is a RNA polymerase I transcription termination protein that also functions as a transcription factor [54]. In our genetic screens, *reb1* deletion cells showed an almost complete lack of growth on respiratory media. The genes repressed in antimycin A-treated cells showed substantial overlaps with the genes repressed in the *rpm1* and *reb1* mutants, while much less overlap was evident among the induced genes (Fig. 7b). These results point to a coordinated regulation of nuclear genes, defining a retrograde response to mitochondrial dysfunction.

Most of the genes that were repressed in the retrograde response encode mitochondrial proteins, most notably components of the ETC and ATP synthase complex (Fig. 7c, d). Block or dysfunction of the ETC leads to increased production of reactive oxygen species, and the down-regulation of OXPHOS genes can protect cells by reducing oxidative damage. Surprisingly, genes encoding the respiratory complex 2 were not repressed in the three data sets (Fig. 7d). The succinate dehydrogenase of complex 2 is part of the TCA cycle; it may therefore be necessary to maintain complex 2 activity because metabolites produced by the TCA cycle are important for anabolic reactions.

The 28 genes induced during the retrograde response (Fig. 7c) were enriched for CESR and oxidative stress (*P* <9.0E-10 and <1.7E-8, respectively). The induced retrograde response also included nine genes which encode oxidoreductases, some of which have poorly understood functions. Increased fermentation might lead to an accumulation of NADH in the cytoplasm [55], and up-regulation of cytoplasmic oxidoreductases could help to stabilise the cellular redox balance. The high-affinity glucose transporter gene *ght5* [56] was also induced (Fig. 7c). These results are consistent with the higher glucose consumption observed in antimycin A-treated cells (Fig. 7a). When the carbon flux is restricted to cytoplasmic glycolysis, cells need to utilise more glucose to provide similar amounts of ATP for supporting growth rates similar to untreated cells. Taken together, we define here a retrograde response in fission yeast. This response involves both the repression and induction of distinct, functionally coherent groups of genes that together may ameliorate the effects of mitochondrial damage.

## Discussion

We investigated energy metabolism of fission yeast using complementary functional and expression profiling approaches. We screened a non-essential deletion library for mutants with deficient respiratory growth, compared the transcriptomes of cells proliferating under steady-state fermentative and respiratory conditions, analysed the dynamic changes in gene expression during adaptation to respiratory conditions and identified critical enzymes for acetyl-CoA production and genes regulated in response to mitochondrial dysfunction.

Only few genes were both differentially expressed on respiratory media and also required for respiratory growth (Fig. 3a). This finding is consistent with results in budding yeast showing that genes that are differentially expressed under a given condition overlap only little with the corresponding mutants that show phenotypes under this condition [57]. While genetic screens tend to uncover response regulators, expression profiling typically identifies metabolic pathways and responses. Accordingly, the changes in transcript levels as a function of different carbon sources reflect predicted changes in energy metabolism (Fig. 4). These coherent changes fit the expected metabolic differences between fermenting and respiring cells very well [2, 5], and expression profiling can thus serve to probe cellular metabolic states. The higher uptake of intermediary metabolites for catabolic processes in fermenting cells, together with more rapid proliferation, resembles the metabolic changes in cancer cells for which it can serve as a basic model system [58].

The manipulation of energy metabolism using different carbon sources has been used to assay mitochondrial function and to screen for respiratory mutants in budding yeast [15]. Different types of growth media, even with identical carbon source, can also affect energy metabolism and need to be carefully considered for such experiments (Fig. 1). Our results, supported by literature evidence, provide a general overview of the genetic and regulatory basis of energy metabolism in fission yeast. The genetic basis for respiratory growth appears to be remarkably distinct between fission and budding yeast: we uncovered 154 genes that are important for respiratory growth in *S. pombe* but whose orthologs have not been identified in corresponding *S. cerevisiae* screens or which do not have orthologs in *S. cerevisiae*. Out of these 154 genes, 92 are conserved in metazoa, with at least 15 reported to be associated with human diseases [59]. Fission yeast thus provides a valuable complementary model system to associate energy metabolism with basic cellular function. On the other hand, the genes being differentially expressed as a function of energy metabolism showed much higher overall concordance between the two yeasts: for example, ~50% of the *S. pombe* genes regulated in glycerol are also regulated during the *S. cerevisiae* post-diauxic shift [5] (Additional file 2: Figure S14). This finding is in accordance with other processes, like the cellular stress response [35], where regulatory mechanisms evolve more rapidly than the genes being regulated.

Our genetic screens using both auxotroph and prototroph mutant libraries uncovered strong genetic interactions between the auxotroph mutants (*ade6, leu1* or *ura4*) and deletion mutants affecting respiratory function. A large number of mutants were required for respiratory growth specifically in either the auxotroph or prototroph backgrounds (Fig. 2b). For example, the mutants only identified in the auxotroph background require the presence of auxotroph mutants for the respiratory phenotype to manifest, pointing to negative genetic interactions with the auxotroph markers. The *ura4* deletion mutant, defective in uracil synthesis, is likely the main cause of this effect for the following reasons: 1) this deletion results in decreased growth on glycerol [24]; 2) the pyrimidine synthesis pathway is linked to reduction of coenzyme Q which may directly impact the ETC and antioxidant defence [49, 60]; and 3) this deletion affects cell wall integrity [61], which could indirectly compromise respiratory metabolism. Such genetic interactions may complicate functional analyses of the corresponding respiratory genes. The distinct results obtained from mutant libraries differing in their genetic background highlight the importance of considering effects from auxotrophies, especially when studying metabolic processes. This point is also highlighted by a recent report of extensive gene expression epistasis as a function of the metabolic-genetic background in *S. cerevisiae* strains [31]. Here we obtained valuable complementary insights into the genetics of energy metabolism by using both auxotroph and prototroph libraries.

The shift from fermentation to respiration is controlled by multiple pathways. The glucose-sensing Pka1 pathway is repressed during respiration [11]. Our RNA-seq data, however, showed that transcripts functioning in the Pka1 pathway are slightly higher expressed in respiratory than in fermentative conditions (Additional file 2: Figure S15). This finding could reflect a sensitization, in that cells prepare to rapidly return to fermentation when conditions allow. We also found that deletion of *tor1* (functioning in the TORC2 complex) inhibits respiratory growth, adding to recent evidence that TORC2 is involved in the regulation of carbon metabolism [39, 62].

Genes encoding the two transcription factors Rsv1 and Rsv2 were strongly induced in response to respiratory conditions; their orthologs in budding yeast (Mig1–3) are implicated in glucose repression [4]. The *rsv1* deletion was missing from our deletion library, and the *rsv2* deletion did not affect respiratory growth. Rsv1 is required to maintain viability under glucose depletion during stationary phase [63]. Rsv2 has been shown to induce stress-related genes during spore formation, while Rsv1 represses glucose metabolism genes [64]. The *scr1* gene, encoding a transcription factor related to Rsv1/2, was also induced in response to respiratory conditions in our experiments, consistent with data showing that *scr1* is induced in response to glucose starvation [37]; Scr1 is regulated by the Ssp2 kinase and involved in glucose derepression [65]. Php3 is another transcriptional regulator involved in energy metabolism based on our data, as it was required for respiratory growth. Php3 is a component of the CCAAT-binding complex, which regulates the glucose-repressible *fbp1* gene in *S. pombe* [66]. Accordingly, the orthologous complex in budding yeast (Hap2–5) acts as the main activator of respiratory genes [4]. The Reb1 transcription factor [54] was also required for respiratory growth, consistent with findings that it functions as an activator of nuclear-encoded respiratory genes (M. R.-L., unpublished data). The transcripts for several other transcription factors were induced during respiratory conditions, which may include additional regulators of energy metabolism (Additional file 1: Tables S2 and S3).

**Fig. 8:**
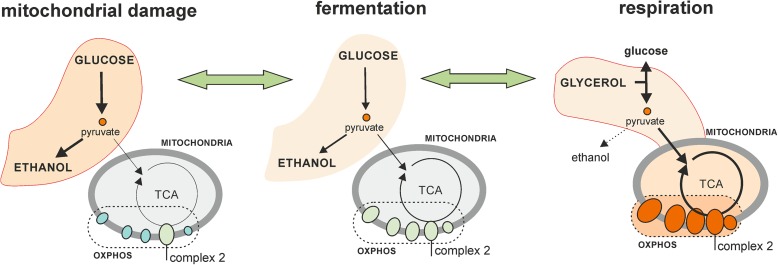
Changes in carbon flow and ETC activity in three biological conditions investigated in this study. Cells grown on glucose gain energy mainly by fermentation (*middle*), but our data indicate that the ETC is also active in this situation and energy gained in respiration process contributes to final biomass production in YES media (Fig. 7a). Cells grown on glycerol gain energy mainly by respiration (*right*), reflected in the up-regulation of ETC transcripts presumably resulting in higher ETC activity. Signals from mitochondrial damage results in repression of the ETC components, except for complex 2, which is also a part of the TCA cycle required for the synthesis of essential biomolecules. In this situation, cells gain all their energy through fermentation, which results in lower biomass production (Fig. 7a)

The transcriptome adaptation to respiratory conditions and the identified retrograde response both involve the coordinated control of OXPHOS genes (Fig. 8). The retrograde response has been intensively studied in budding yeast, where the transcription factors Rtg1 and Rtg2 activate genes of the glyoxylate pathway and part of the TCA cycle to maintain cellular glutamate homeostasis [67]. The glyoxylate pathway is not present in fission yeast, as is also the case in metazoa. Mitochondrial damage in fission yeast led to repression of genes encoding OXPHOS complex proteins, except for complex 2 (succinate dehydrogenase) that also participates in the TCA cycle and may be required to maintain TCA activity (Fig. 8). The TCA cycle could also be required during rapid growth, even when ATP production by OXPHOS is not necessary, to maintain synthesis of biomolecules such as glutamate or aspartate. A recent study has reported that an essential function of respiration in proliferating cells is to support aspartate synthesis [68, 69]; minimal flow through the TCA cycle therefore needs to be maintained even when the ETC is repressed. Iron deficiency also leads to repression of transcripts encoding ETC subunits, but in this condition the succinate dehydrogenase complex is also strongly repressed [70, 71] (Additional file 2: Figure S16). The CCAAT-binding factor Php4 is involved in gene repression upon iron starvation [70]. Php4 is therefore a likely candidate to repress the ETC genes also in response to mitochondrial damage. Anaerobic conditions that also lead to inhibition of respiration result in a similar repression of ETC transcripts [72]. Further work will be required to investigate the signals and transcription factors regulating the retrograde response and related processes.

Our genetic screens identified several chromatin proteins that are involved in respiratory growth, including the argonaute silencing factor Ago1 [73] and the chromatin remodelling complex subunits Rsc1 (RSC complex) [74] and Ies2 (Ino80 complex). Moreover, we detected several clusters of co-regulated genes in respiratory medium (Fig. 3e), and the chromatin silencing factors Cid12 and Set3 were required for respiratory growth on galactose (Additional file 2: Figure S9). These findings suggest that changes in chromatin states are important in regulating the metabolic shift between fermentation and respiration.

We also uncovered several RNA-binding proteins in our genetic screens as being important for respiratory growth. Examples are Mlo3 [75], the polyA-binding protein Nab2 [76] and Mcp2, an ortholog of budding yeast Puf3 that regulates the translation and stability of mRNAs encoding mitochondrial proteins [77]. These results indicate that post-transcriptional levels of regulation play important roles in the control of energy metabolism.

Respiration is essential for meiotic differentiation [16]. Our data indicate that the link between these two processes could be hardwired, in that respiration may even be sufficient to trigger meiotic differentiation. Under respiratory conditions, the meiotic gene expression program was induced and cells managed to efficiently undergo meiosis and sporulation on yeast extract media containing glycerol (Additional file 2: Figure S5). This is a surprising finding as yeast extract is normally a strong repressor of meiotic differentiation in fission yeast.

In multicellular eukaryotes, the ATP citrate lyase activity generates acetyl-CoA used for histone acetylation and thus epigenetically links gene expression with glucose metabolism [42, 78]. The citrate lyase is absent in budding yeast. It has not been known how carbon metabolism is linked to acetyl-CoA synthesis in fission yeast. We show here that the acetyl-CoA synthase Acs1, rather than the citrate lyase, is an essential player in cellular acetyl-CoA synthesis, as is the case in budding yeast [79]. The acetyl-CoA synthase is instrumental for the growth of cancer cells [80, 81], further illustrating the similarities between yeast and cancer metabolism. Further work will be required to test whether the fission yeast citrate lyase has any specialised role in acetyl-CoA metabolism.

## Conclusions

We provide a systematic survey of genes that are required for respiration and analyse gene regulation during the switch from fermentation to respiration. These two sets of genes show remarkably little overlap and provide complementary insights into the functional richness and intricate regulation of energy metabolism. We also study aspects of acetyl-CoA metabolism and define the retrograde response to prevent damage from dysfunctional mitochondria in fission yeast. Our analyses provide rich information on metabolic processes and also serve as a framework for future research on energy metabolism and crosstalk between respiration and other cellular processes.

## Methods

### Yeast strains and growth media

Strains used in this study are listed in Additional file 1: Table S5. For the genetic screening, the auxotroph Bioneer library v2.0 [30] or its prototroph derivative [32] was used. Cells were grown in rich yeast extract (YE) medium with 3% glucose (fermentative medium) or 2% glucose (Fig. 1) or in minimal (EMM) medium with 2% glucose. For respiratory media, YE medium was supplemented with both 3% glycerol and 0.1% glucose or with 2% galactose and 0.1% glucose. For screening of respiratory deficient mutants, solid YE medium was additionally supplemented with adenine, uracil, leucine, histidine and lysine (YES). Where indicated, media were supplemented with antimycin A (0.1 ng/ml for genetic screening or 0.15 μg/ml for inhibition of respiration) or with 2,4-dinitrophenol (1 μg/ml) [21]. For cell mating, malt extract agar (MEA) medium was used.

### Genetic screens for respiratory mutants

The Bioneer haploid deletion mutant library v2.0 (3005 mutants) or prototroph library (2847 strains) was arrayed using a RoToR HDA robot (Singer Instruments) onto solid YES media in 1536 format, with each mutant spotted in quadruplicate. Subsequently, arrays were copied onto fermentative and respiratory media. Plates were incubated at 32 °C for 2 days, and images were acquired using a Canon camera and multidoc imagining system (UVP). Quantification of colony sizes was performed with the gitter R package [82]. Colony sizes were normalised to the median colony size of the plate, and colony sizes for each mutant strain were calculated as a median of four replicate colonies analysed per mutant. Subsequently colony size ratios of strains grown on respiratory relative to fermentative media were calculated.

The colony size ratios calculated for the different screens (Additional file 1: Table S1) were imported into GeneSpring GX13 software (Agilent Technologies). Lower values of colony size ratios were set at a threshold of 0.2. Mutants with data missing for any of the conditions were removed. Using the Self-Organizing Map clustering method with default settings, the mutants were grouped into 12 clusters. GO category enrichments in each cluster were calculated using the AnGeLi web tool [83]. *S. cerevisiae* genes associated with phenotype categories “respiratory growth: decreased rate” and “respiratory growth: absent” were checked for *S. pombe* orthologues using the manually curated orthologue list available in PomBase [59]. This list was then restricted to the strains included in the Bioneer collection v2.0 and compared to genes identified in the screen and to the *S. pombe* phenotype category FYPO:0001934 (“abolished cell growth on glycerol carbon source”) [59].

### RNA sequencing

Wild-type yeast cells (*972 h-*) were grown on YES or YE media with 3% glycerol and 0.1% glucose and harvested at early exponential growth phase (OD 0.5), and total RNA was isolated by hot-phenol extraction [84]. RNA quality was assessed on a Bioanalyzer instrument (Agilent), treated with DNase (Turbo DNA-free, Ambion) and subsequently 4 μg of RNA was treated with a beta version of Ribo-Zero Magnetic Gold Kit Yeast (Epicentre) to deplete rRNAs. RNA-seq libraries were prepared from rRNA-free RNA using a strand-specific library preparation protocol [85] and sequenced on an Illumina HiSeq instrument. Sequence data analysis was carried out as described [85], with the exception of using only annotated regions (7022 annotated genes) and 51-bp reads. The significance of overlapping gene lists was calculated with the hypergeometric probability formula using the *phyper* R function.

### Generation of deletion mutants and tetrad dissection

Diploid strains were selected on EMM media from a cross of *ade6-210 h +* and *ade6-216 h-* strains and afterwards grown on YES media. Diploids were transformed with a deletion cassette for *acs1* containing the hygromycin marker [86, 87]. Positive clones were selected and the deletion junctions were checked by PCR. The diploid strain was then sporulated on MEA medium, and tetrads were dissected on YES medium using a MSM 400 dissection microscope (Singer Instruments). The grown spore colonies were then replicated onto YES medium with hygromycin (0.1 mg/ml).

### Western blotting

Total protein extracts were prepared using the FastPrep-24 equipment (MP) in PBS buffer with protease inhibitors. Protein concentrations of the soluble fractions were adjusted using the BCA Protein Assay (Thermo Scientific). About 10 μg of proteins from the soluble fractions were separated on the NuPAGE 4–12% acrylamide gels (Novex) and transferred to nitrocellulose membranes (mini Trans-Blot Cell BioRad). Antibodies against actin, histone H3 and histone H3K9 (Ambion), and appropriate secondary antibodies, were used according to the manufacturer’s instructions.

### Time course and antimycin A experiments using microarrays

For the time course analyses, cells were grown to early exponential phase (OD 0.5) in fermentative medium, washed once in sterile water and re-suspended in the same volume of respiratory medium. Transcriptomes were analysed before (time point 0) and at six time points after the change of carbon source, up to 24 h. RNA from cell pellets was isolated using hot phenol extraction, followed by labelling of the single samples and a pool of all the samples which served as reference [84]. Agilent 8 × 15 K custom-made *S. pombe* expression microarrays were used, and hybridizations and subsequent washes were performed according to the manufacturer’s protocols. Microarrays were scanned using a GenePix 4000 B laser scanner, and fluorescence signals were analysed using GenePix Pro software (Axon Instruments). The resulting data were processed using customized R scripts for quality control and normalization and analysed using GeneSpring GX13 [84, 88]. Two independent biological repeats with a dye swap were performed. The K-means algorithm was used for clustering (Additional file 2: Figure S11).

For investigating the retrograde response, wild-type cells with or without antimycin A treatment were grown in YES medium to OD ~0.5. The RNA of these cells was then isolated and processed for microarray analysis as described above. Differentially labelled RNA from treated versus untreated cells was analysed from three independent biological repeats including a dye swap.

### Determining glucose and ethanol concentration in media

Cells were grown in YES medium with or without antimycin A (0.15 μg/ml). At the indicated time points, 1 ml of cell culture was precipitated, and the supernatant was assayed for ethanol and glucose concentrations using the Ethanol Assay Kit (Abcam) or Glucose (HK) Assay (Sigma), respectively.

## Additional files

Additional file 1: Supplementary tables. **Table S1.** Colony size ratios from nine genetic screens for respiratory mutants. **Table S2.** Results of RNA-seq analysis of transcriptomes of cells grown on glycerol versus glucose and selected groups of differentially expressed genes. **Table S3.** Relative expression data from microarray profiling after shift to respiratory medium and k-means gene clusters. **Table S4.** List of genes differentially expressed on YES + antimycin A relative to YES. **Table S5.** List of strains used in this study. (XLSX 1837 kb)
Additional file 2: Supplementary figures. **Figure S1.** Heat map of data from genetic screens for respiratory deficient mutants. **Figure S2.** Analysis of 34 deletion mutants that affect growth in prototroph and auxotroph libraries. **Figure S3.** Glycerol growth data from this study of strains annotated elsewhere to impact growth on glycerol (FYPO:0001934). **Figure S4.** Analyses of non-coding RNAs regulated in fermentative versus respiratory conditions. **Figure S5.** Respiratory medium induces abundant cell mating and sporulation. **Figure S6.** Cell growth on different carbon sources. **Figure S7**. Region on chromosome 3 where seven neighbouring genes are induced after shift to respiratory conditions. **Figure S8.** Genes located at ends of chromosome 2 become de-repressed after shift to respiratory conditions. **Figure S9.** Deletion of *set3* and *cid12* specifically affects growth on galactose in prototroph background. **Figure S10.** Mitochondrial network becomes more divided in respiratory conditions. **Figure S11.** Clustering of genes that change expression during adaptation to respiratory growth. **Figure S12.** Genes encoding ETC proteins and ATP synthase components are induced in distinct, coordinated ways during adaptation to respiratory growth. **Figure S13.** Changes in transcript levels during adaptation to respiratory growth for genes important for energy metabolism. **Figure S14.** Overlap between *S. pombe* genes induced on glycerol (RNA-seq data) and *S. cerevisiae* genes induced upon diauxic shift (only genes with orthologs in both species are considered). **Figure S15.** Changes in expression of genes encoding key elements of glucose signalling pathways. **Figure S16.** Venn diagrams showing overlaps between retrograde response genes and genes regulated in response to iron depletion, oxidative stress or CESR genes. (PDF 7252 kb)
