# Supplementary material for: Functional and regulatory profiling of energy metabolism in fission yeast
Source: Genome Biol. 2016 Nov 25;17:240. doi: 10.1186/s13059-016-1101-2 (PMC5124322; doi:10.1186/s13059-016-1101-2)
Supplement: Additional file 2: — Supplementary figures. Figure S1. Heat map of data from genetic screens for respiratory deficient mutants. Figure S2. Analysis of 34 deletion mutants that affect growth in prototroph and auxotroph libraries. Figure S3. Glycerol growth data from this study of strains annotated elsewhere to impact growth on glycerol (FYPO:0001934). Figure S4. Analyses of non-coding RNAs regulated in fermentative versus respiratory conditions. Figure S5. Respiratory medium induces abundant cell mating and sporulation. Figure S6. Cell growth on different carbon sources. Figure S7. Region on chromosome 3 where seven neighbouring genes are induced after shift to respiratory conditions. Figure S8. Genes located at ends of chromosome 2 become de-repressed after shift to respiratory conditions. Figure S9. Deletion of set3 and cid12 specifically affects growth on galactose in prototroph background. Figure S10. Mitochondrial network becomes more divided in respiratory conditions. Figure S11. Clustering of genes that change expression during adaptation to respiratory growth. Figure S12. Genes encoding ETC proteins and ATP synthase components are induced in distinct, coordinated ways during adaptation to respiratory growth. Figure S13. Changes in transcript levels during adaptation to respiratory growth for genes important for energy metabolism. Figure S14. Overlap between S. pombe genes induced on glycerol (RNA-seq data) and S. cerevisiae genes induced upon diauxic shift (only genes with orthologs in both species are considered). Figure S15. Changes in expression of genes encoding key elements of glucose signalling pathways. Figure S16. Venn diagrams showing overlaps between retrograde response genes and genes regulated in response to iron depletion, oxidative stress or CESR genes. (PDF 7252 kb) [file 13059_2016_1101_MOESM2_ESM.pdf]

Algorithm: Self-Organizing Map
Parameters:
Cluster on Rows
Distance metric = Euclidean
Max num of iterations = 50
Num of grid rows = 3
Number of grid col = 4
Initial learning rate = 0.03
Initial neighbourhood radius = 5
Grid topology = Hexagonal
Neighbourhood type = Bubble
Software used - GeneSpring 13.0

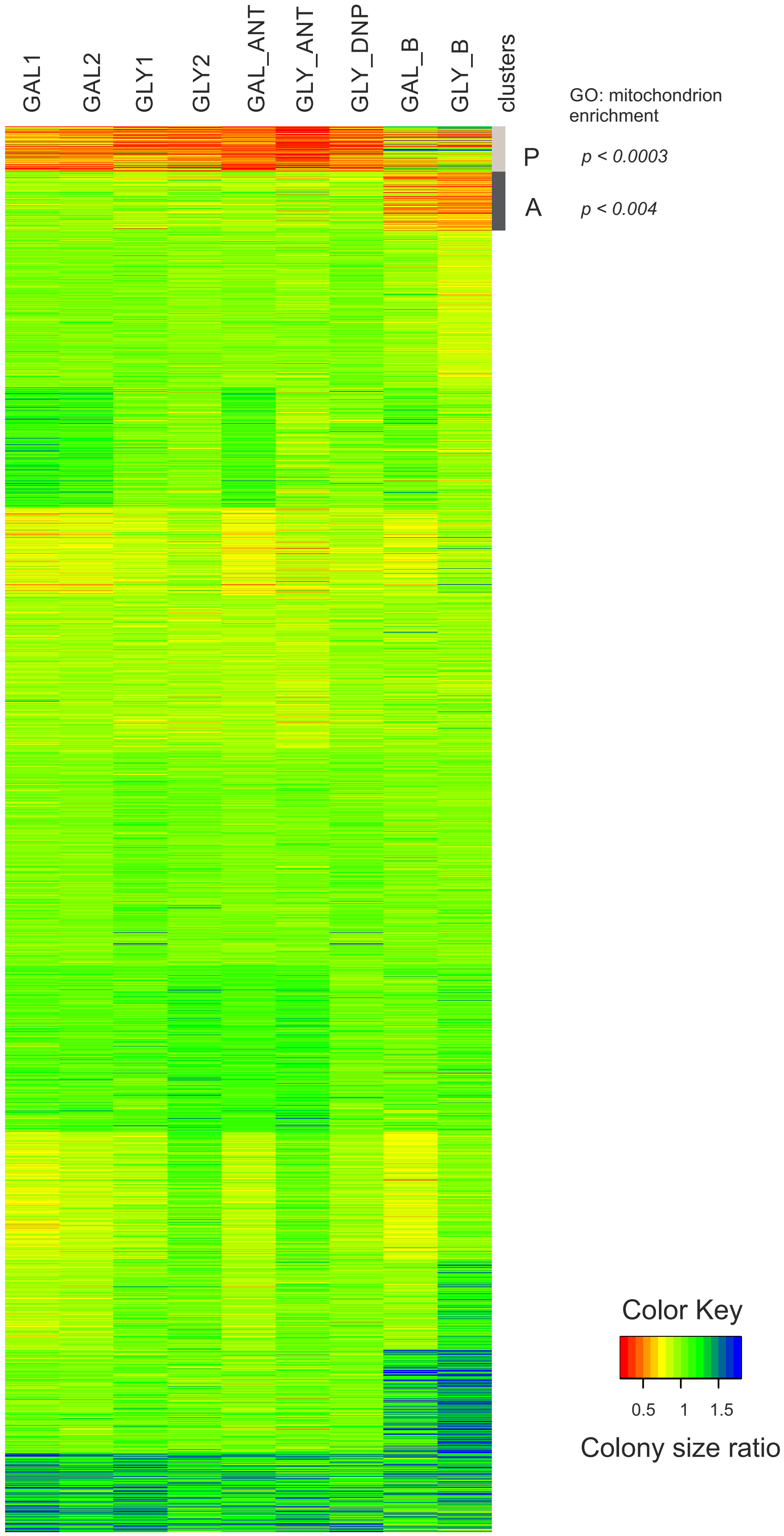

S1 Fig. Heat map of data from genetic screens for respiratory deficient mutants. Colony size ratios on respiratory conditions relative to fermentative control, collected for all mutants across all samples, clustered using a self-organizing map algorithm. Genes in cluster order were exported, and the heat map was visualized using the heatmap2 R package. Clusters P and A are indicated at right. Both of these clusters are significantly enriched for genes encoding mitochondrial proteins (Cluster A, $p < 0.004$; Cluster P, $p < 0.0003$) [83].

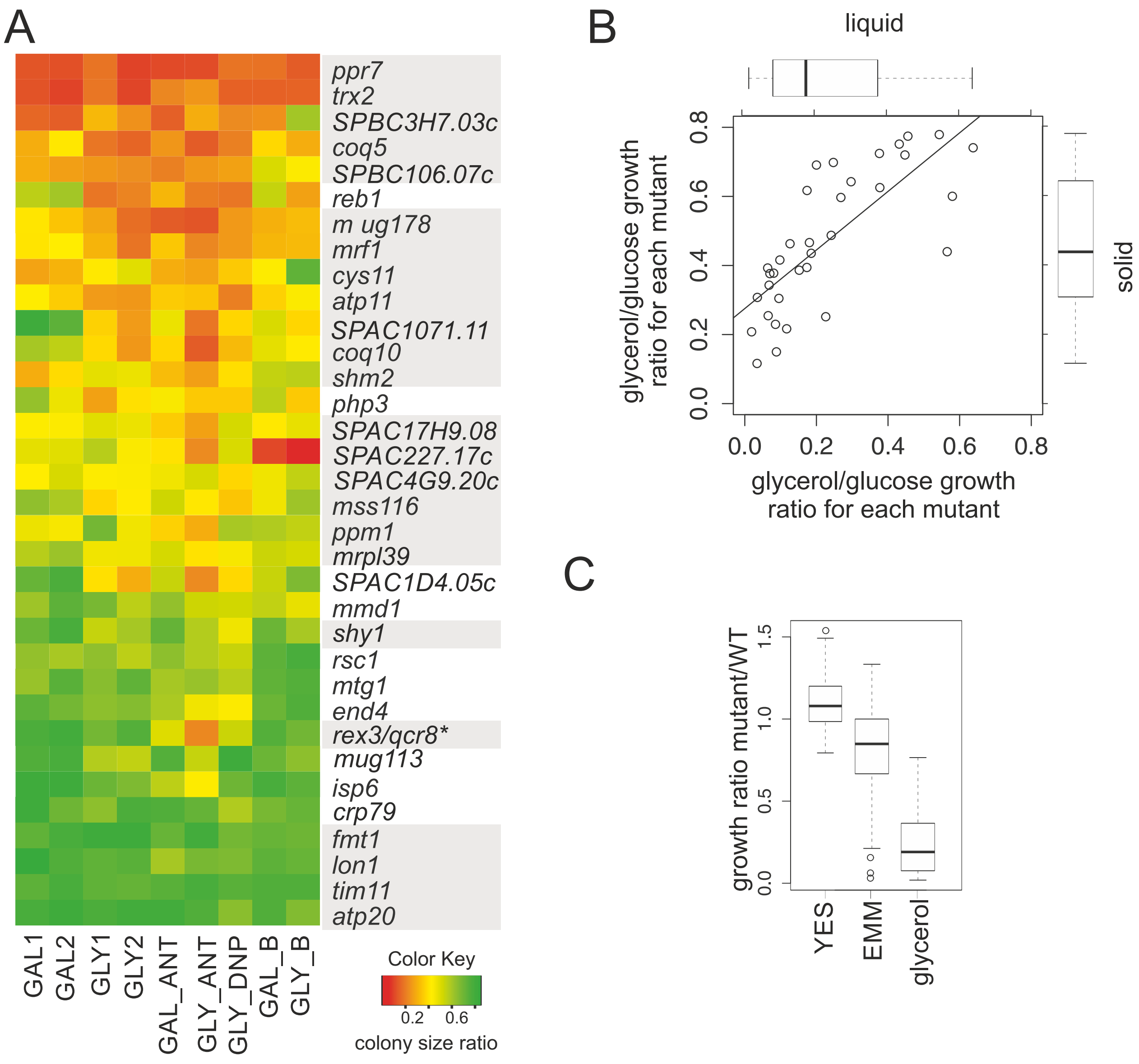

* deletion of *rex3* influences expression of *qcr8*, which is most probable cause of respiratory phenotype of this mutant (data not shown)

S2 Fig. Analysis of 34 deletion mutants that affect growth in prototroph and auxotroph libraries. (A) Heat map represents colony size ratios on respiratory conditions relative to fermentative control for each experiment performed. (B) Median colony size ratios from genetic screens for the 34 mutants plotted against corresponding ratios of maximal growth curve slopes of these mutants in liquid respiratory vs fermentative media. (C) Boxplot showing maximal growth curve slope ratios for the 34 mutants relative to wild-type controls when grown on three different media as indicated. Growth of respiratory mutants is slower in minimal medium (EMM) than in rich medium (YE), and is strongly inhibited in respiratory medium (glycerol).

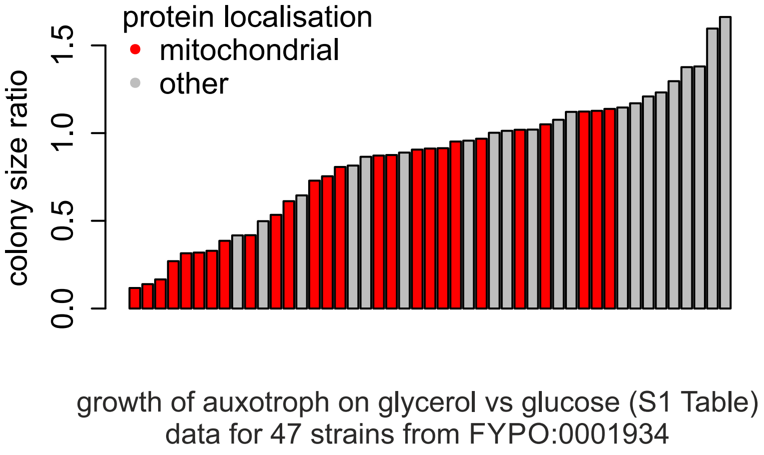

S3 Fig. Glycerol growth data from this study of strains annotated elsewhere to impact growth on glycerol (FYPO:0001934). Mitochondrial localisation of protein encoded by deleted gene is indicated by red colour.

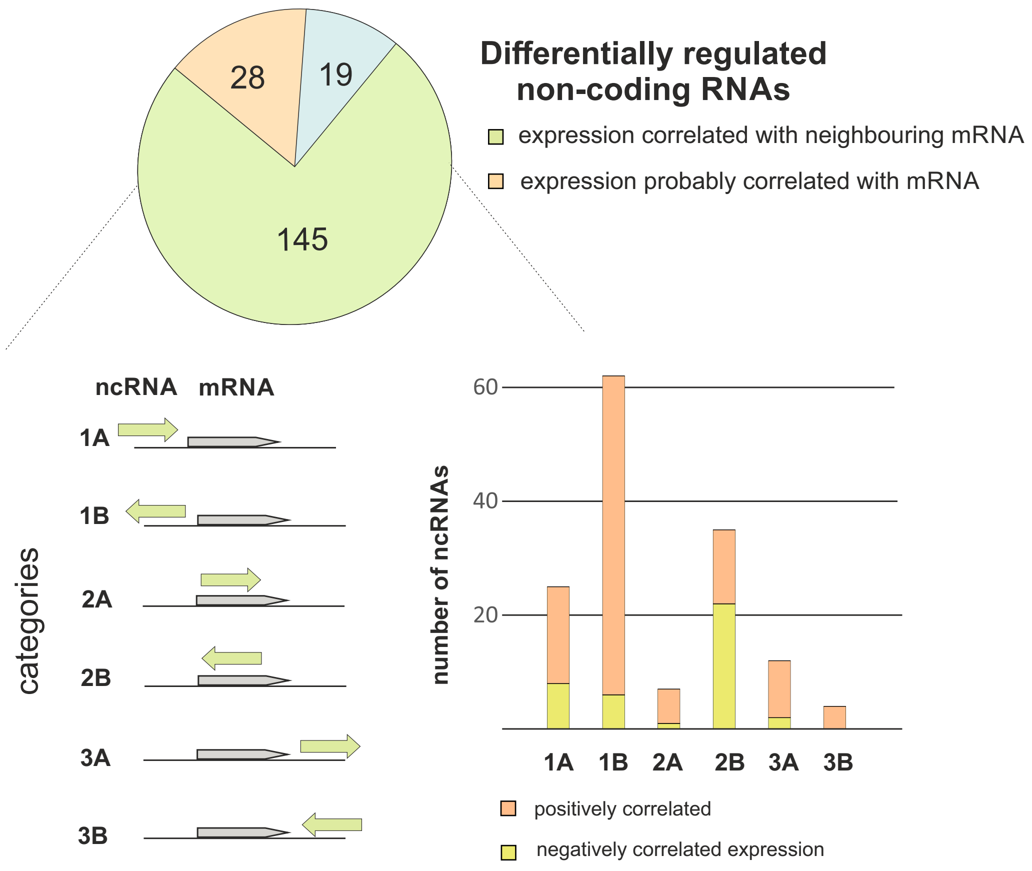

S4 Fig. Analyses of non-coding RNAs regulated in fermentative vs respiratory conditions. Out of 192 differentially regulated ncRNAs, 145 have neighbouring genes whose expression changes significantly ($p < 0.05$). Among the remaining 47 ncRNAs, a further 28 have neighbouring genes whose expression changes, albeit below our threshold of significance. The group of 145 ncRNAs is further divided into categories, depending on the positions and directions of neighbouring genes. The bar plot shows fractions of gene pairs whose expression correlates positively or negatively for each category.

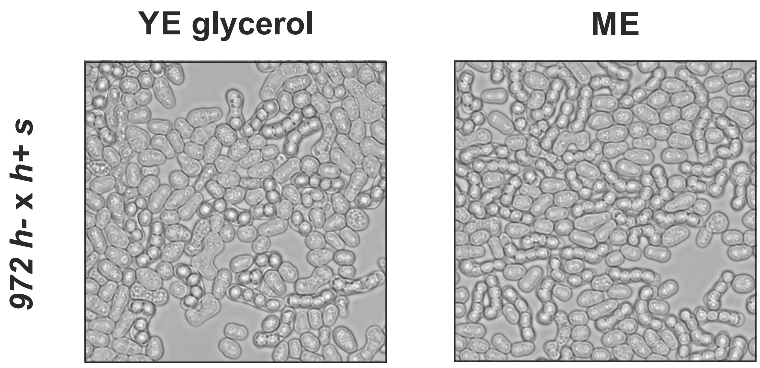

S5 Fig. Respiratory medium induces abundant cell mating and sporulation. Yeast cells of opposite mating types grown on minimal media, mixed and plated on either malt extract (MEA) or yeast extract plates containing 3% glycerol and 0.1% glucose (YES glycerol). Pictures taken after two days of incubation at 25^{0}C.

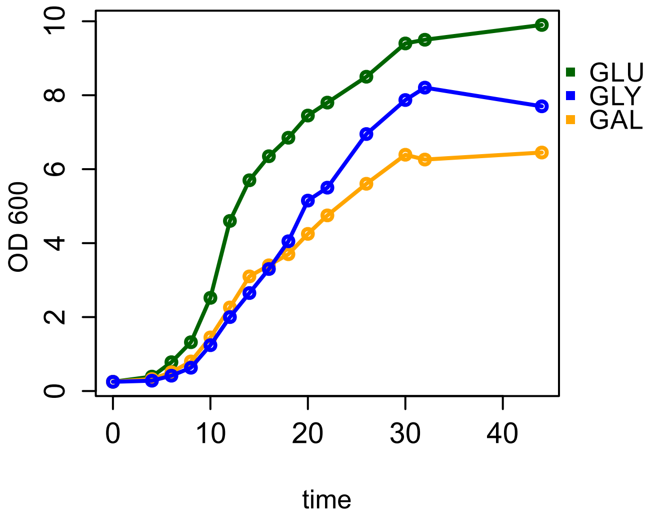

S6 Fig. Cell growth on different carbon sources. Growth curves recorded for batch cultures grown in YE glucose (GLU), YE glycerol (GLY) or YE galactose (GAL) media.

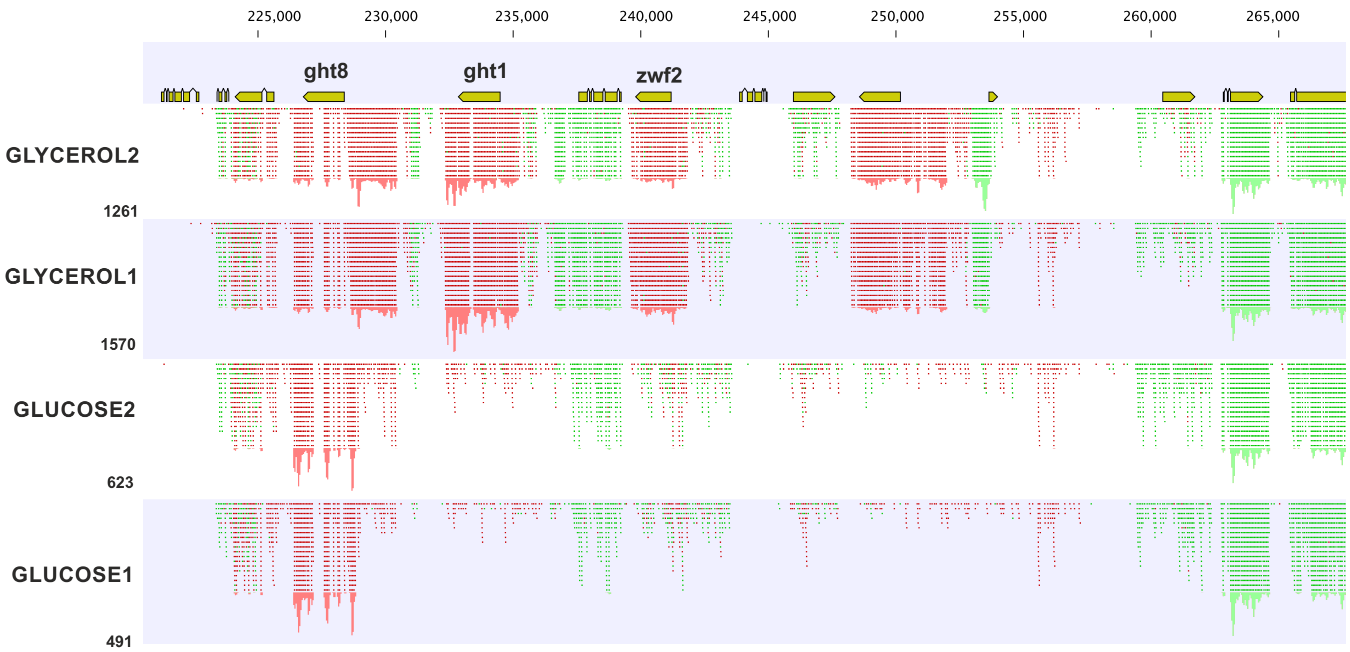

S7 Fig. Region on chromosome 3 where 7 neighbouring genes are induced after shift to respiratory conditions. RNA-seq read coverage along chromosome for cells grown on glucose or glycerol media (two biological repeats each), with read directionality depicted by red (forward) and green (reverse) colours. Genes known to be involved in glucose metabolism are named. Graph prepared using CLC Genomics software.

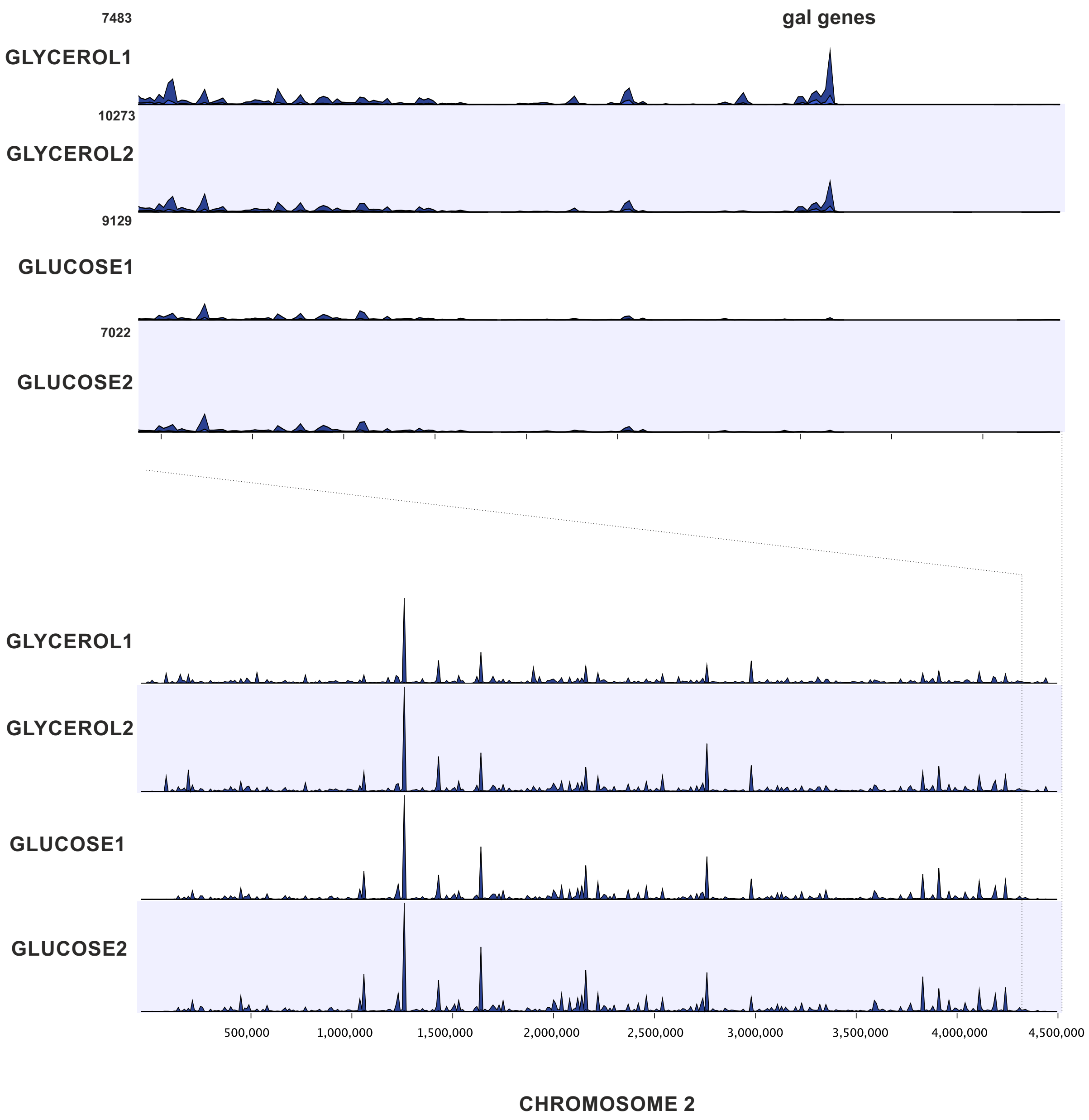

S8 Fig. Genes located at ends of chromosome 2 become de-repressed after shift to respiratory conditions. RNA-seq read coverage along chromosome for cells grown on glucose or glycerol media (two biological repeats each). The upper part shows zoomed section of right arm of chromosome 2, with positions of galactose metabolism genes indicated. Graph prepared using CLC Genomics software.

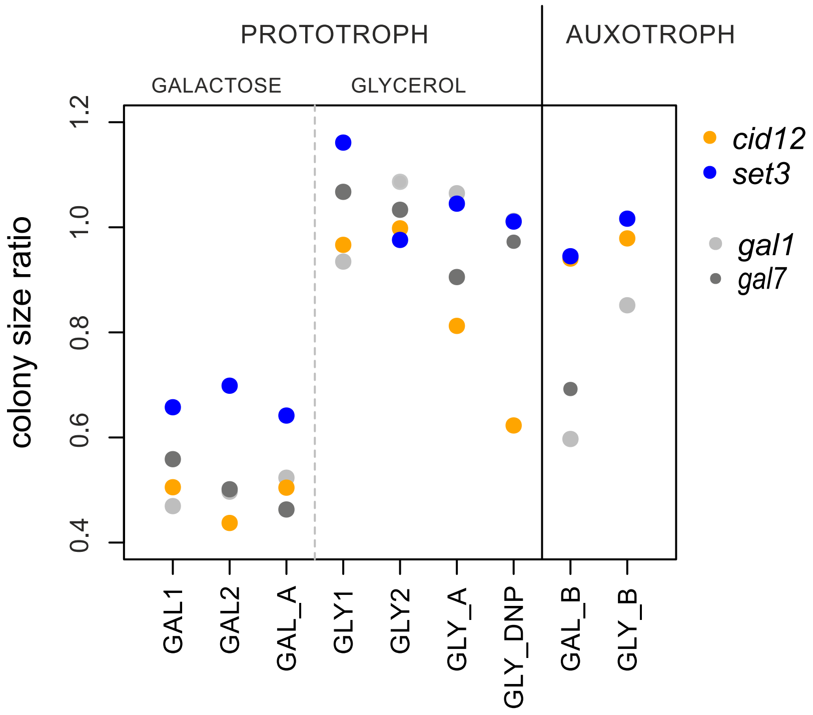

S9 Fig. Deletion of *set3* and *cid12* specifically affects growth on galactose in prototroph background. Colony size ratios across different genetic screens are indicated for 4 mutants, with *gal1* and *gal7* deletions serving as controls (genes with known function in galactose metabolism). Data are given in Additional file 1: S1 Table.

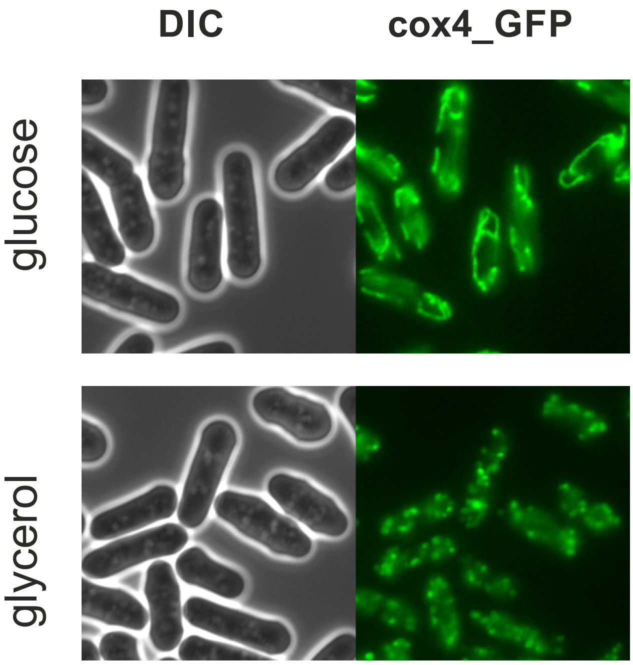

S10 Fig. Mitochondrial network becomes more divided in respiratory conditions. Cells with *cox4* gene endogenously tagged with GFP grown to early exponential phase in glucose or glycerol media as indicated, and Cox4-GFP protein distribution visualised using fluorescence microscopy.

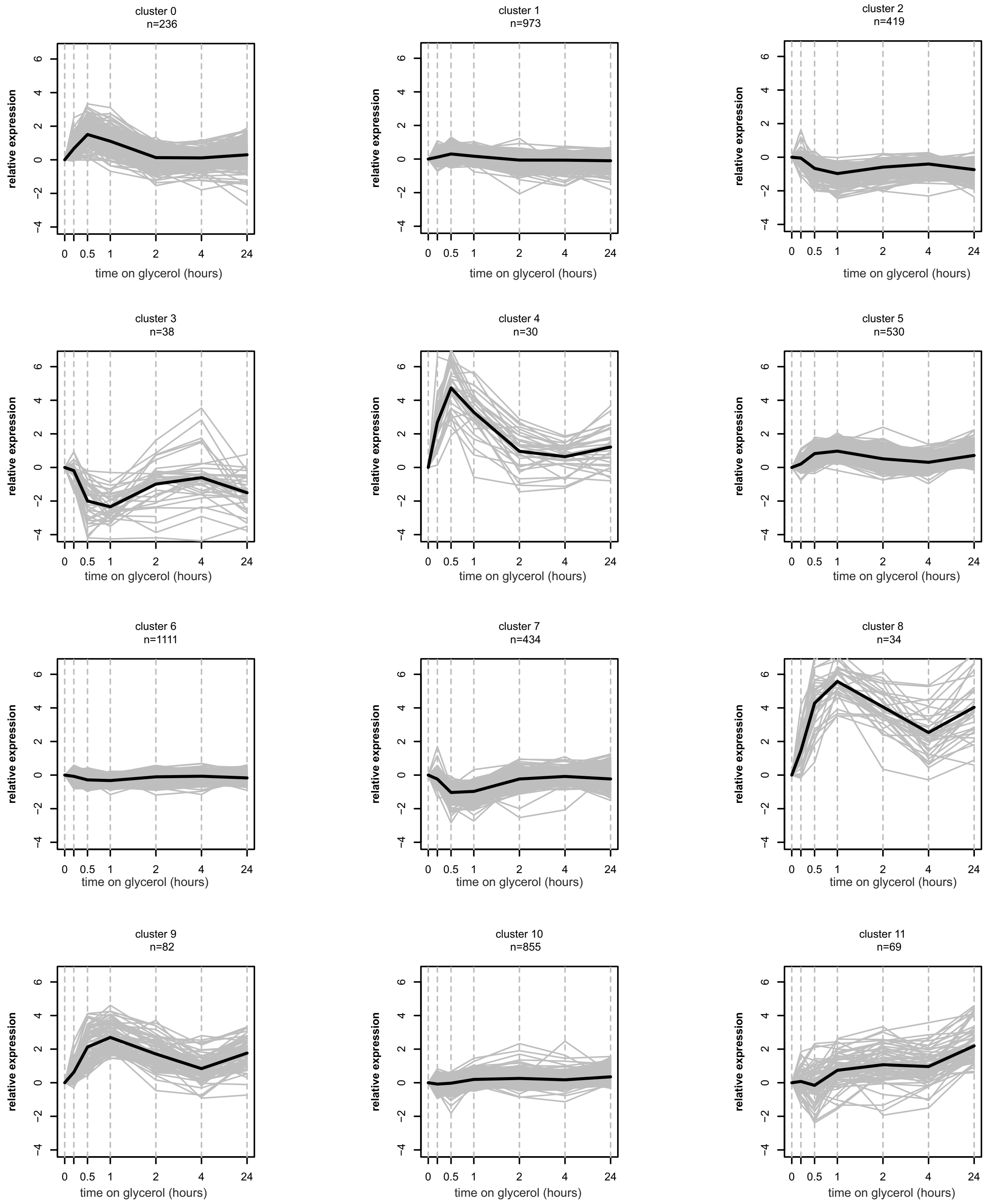

S11 Fig. Clustering of genes that change expression during adaptation to respiratory growth. All genes clustered into 12 groups using k-means. Gene expression profiles for each cluster depicted as grey lines, with average changes for each cluster shown by black lines. The list of genes for each cluster along with enriched GO categories, if applicable, are given in Additional file 1: S3 Table.

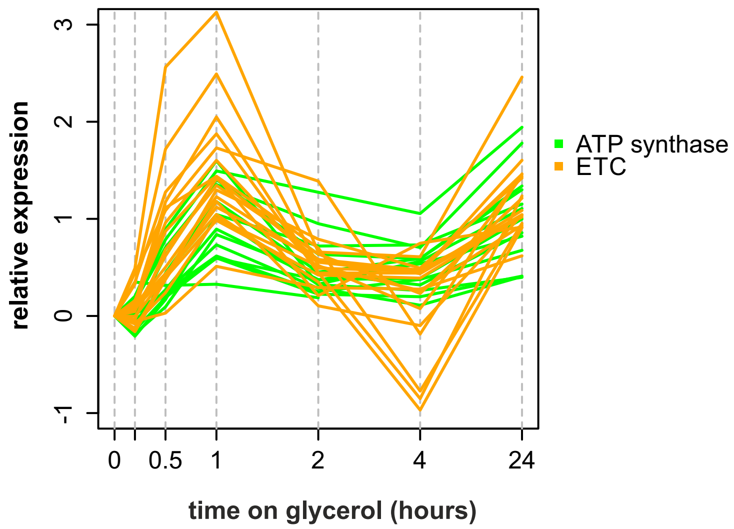

S12 Fig. Genes encoding ETC proteins and ATP synthase components are induced in distinct, coordinated ways during adaptation to respiratory growth.

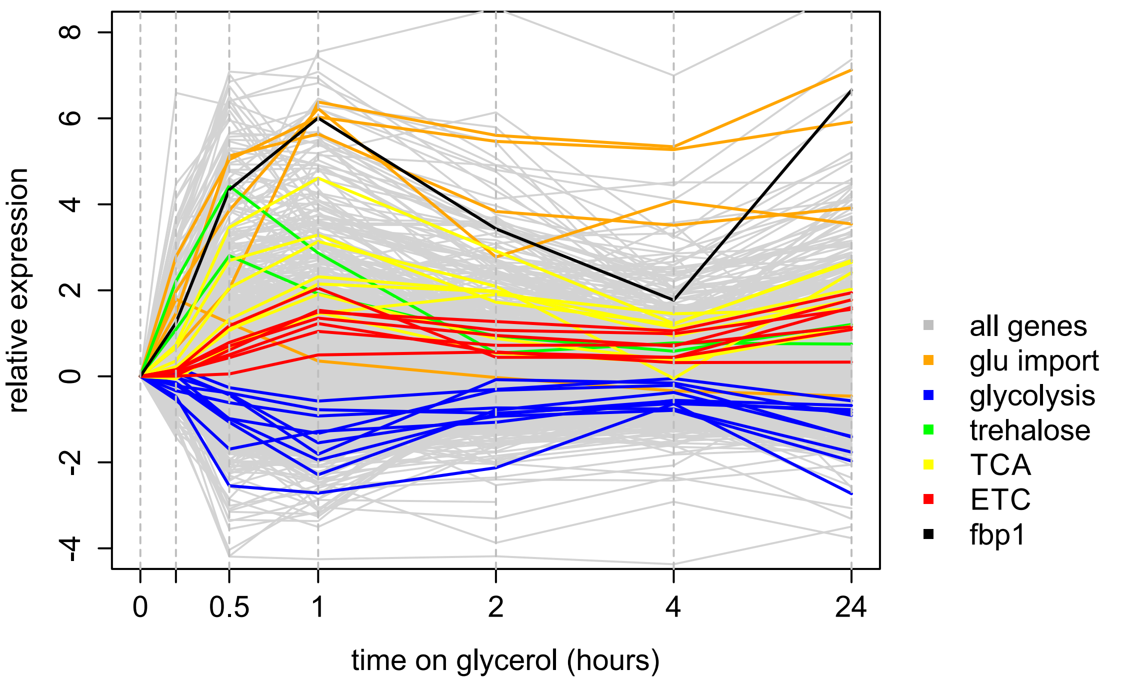

S13 Fig. Changes in transcript levels during adaptation to respiratory growth for genes important for energy metabolism. Genes chosen based on RNA-seq results and coded in same colours as squares next to corresponding genes in Fig 4.

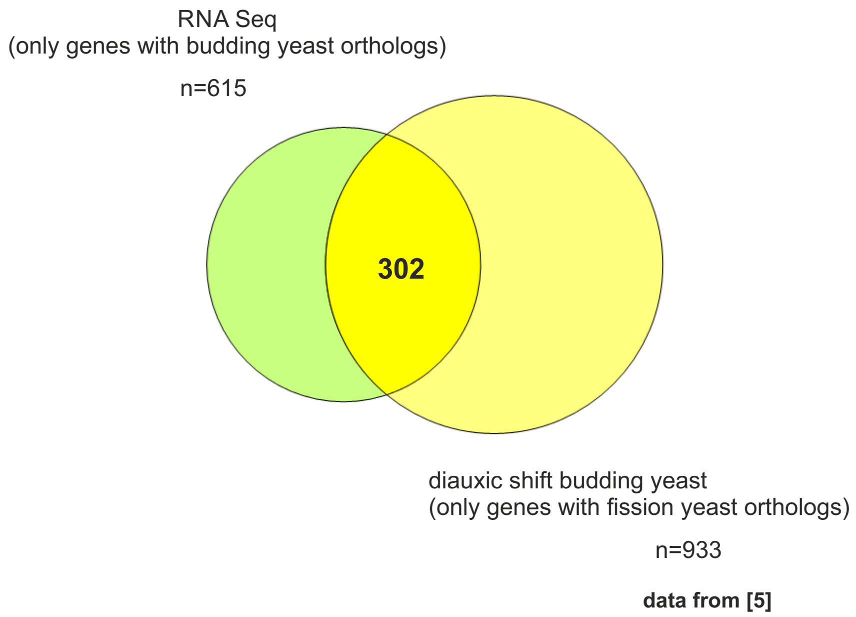

S14 Fig. Overlap between *S. pombe* genes induced on glycerol (RNA seq data) and *S. cerevisiae* genes induced upon diauxic shift (only genes with orthologs in both species are considered).

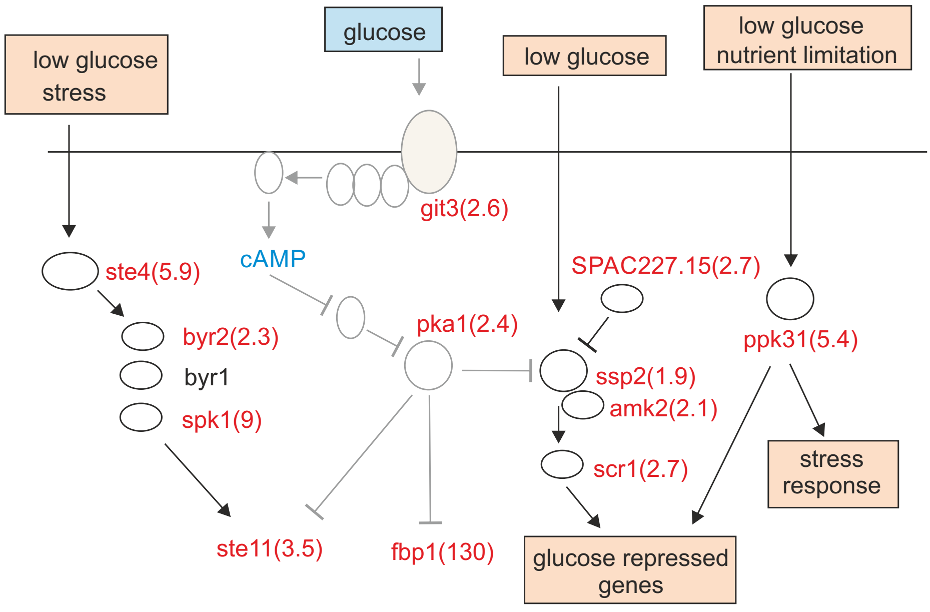

S15 Fig. Changes in expression of genes encoding key elements of glucose signalling pathways. Based on RNA-seq results for cells grown on respiratory vs fermentative conditions (data provided in Additional file 1: S2 Table).

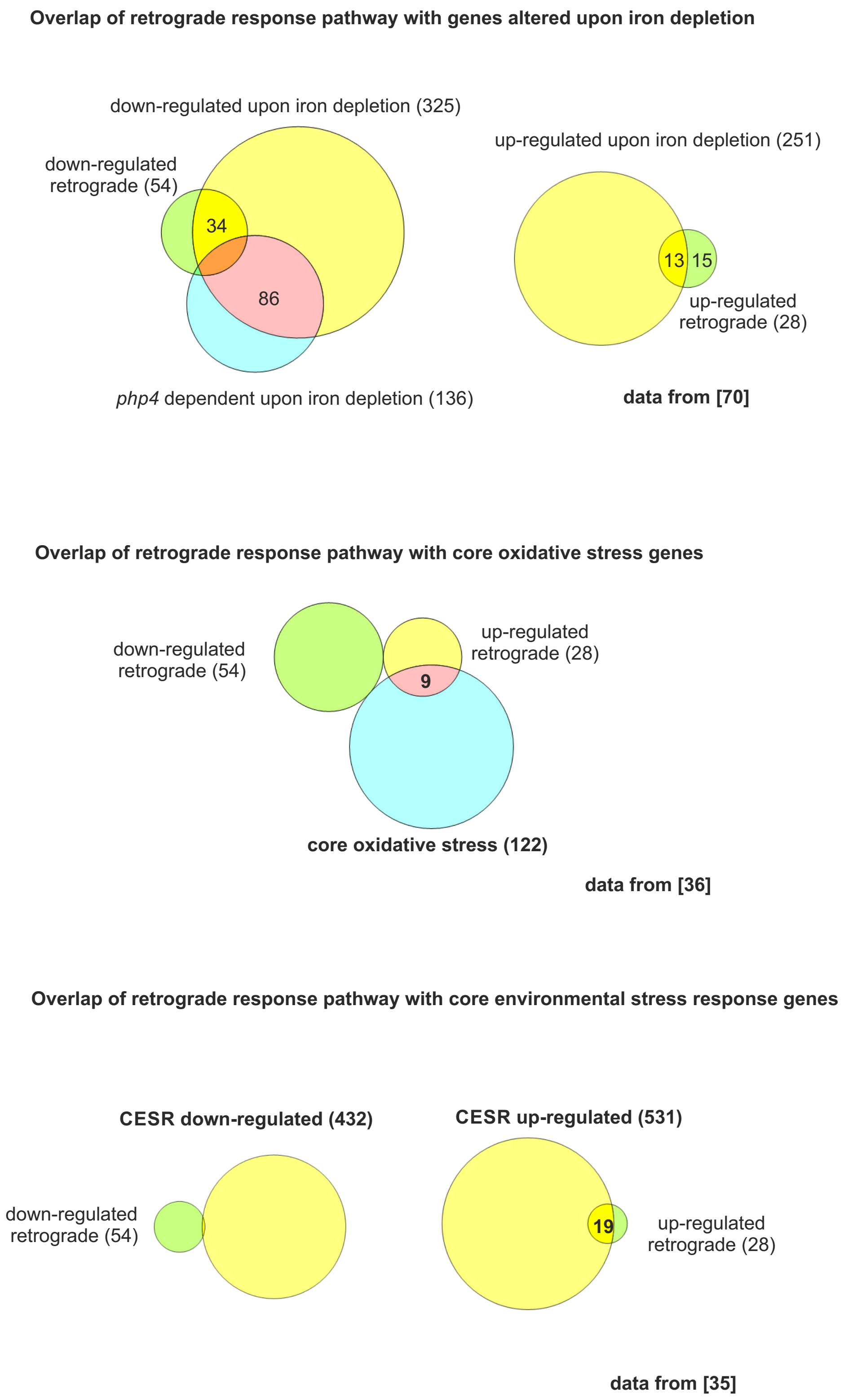

S16 Fig. Venn diagrams showing overlaps between retrograde response genes and genes regulated in response to iron depletion, oxidative stress, or CESR genes.
